# Serum Metabolomics Analysis Reveals a Distinct Metabolic Profile of Patients with Primary Biliary Cholangitis

**DOI:** 10.1038/s41598-017-00944-9

**Published:** 2017-04-11

**Authors:** Juan Hao, Tao Yang, Yang Zhou, Guo-Yuan Gao, Feng Xing, Yuan Peng, Yan-Yan Tao, Cheng-Hai Liu

**Affiliations:** 1 Institute of Liver Diseases, Shuguang Hospital Affiliated to Shanghai University of Traditional Chinese Medicine, 528 Zhangheng Road, Shanghai, 201203 China; 2 Institute of Cardiovascular Disease, Shuguang Hospital Affiliated to Shanghai University of Traditional Chinese Medicine, Shanghai, 201203 China; 3 School of Pharmacy, East China University of Science and Technology, Shanghai, 200237 China; 4 E-Institute of Traditional Chinese Medicine Internal Medicine, Shanghai Municipal Education Commission, 1200 Cailun Road, Shanghai, 201203 China; 5 Shanghai Key Laboratory of Traditional Chinese Clinical Medicine, Shanghai, 201203 China

## Abstract

Primary biliary cholangitis (PBC) is a chronic autoimmune liver disease associated with profound metabolic changes. The purpose of this study was to identify a distinctive metabolic signature from the training set with 29 PBC patients, 30 hepatitis B virus (HBV)-caused cirrhosis (HBC) and 41 healthy controls, and to validate the applicability and stability of the distinctive model from the validation set with 21 PBC patients, 7 autoimmune hepatitis (AIH) and 9 HBC. The sera were investigated using high resolution nuclear magnetic resonance (NMR) and the datasets were analyzed pairwise using pattern recognition methods. 45 distinguishable metabolites were identified and 15 metabolic pathways were reprogrammed. The altered metabolic pathways were associated with glucose, fatty acid and amino acid metabolites. Logistic regression and ROC analysis were used to establish a diagnostic model with the equated (p) = −12.22–3.46*log(4-hydroxyproline) + 6.62*log(3-hydroxyisovalerate) − 2.44*log(citraconate) − 3.80*log(pyruvate). The area under the curve (AUC) of the optimized model was 0.937 (95% confidence interval (CI): 0.868–0.976) in the training set and 0.890 (95% CI: 0.743–0.969) in the validation set. These results not only revealed the potential pathogenesis of PBC, but also provided a feasible diagnostic tool for PBC populations through detection of serum metabolites.

## Introduction

Primary biliary cholangitis (PBC), which until recently was named primary biliary cirrhosis, is a chronic autoimmune liver disease characterized by the progressive destruction of small intrahepatic bile ducts that can lead to chronic cholestasis, portal fibrosis, and cirrhosis^1^. A population-based epidemiological study revealed prevalence rates ranging from 1.91 to 40.2 per 100,000 people that appeared to increase over time^2, 3^. The etiology and pathogenesis of PBC are poorly understood, although the disease is characterized by a female predominance and anti-mitochondrial antibodies (AMAs) and is associated with autoimmune syndromes and histological features of chronic inflammation^4^.

The natural history of PBC varies greatly between patients, ranging from asymptomatic and slowly progressive to a symptomatic and rapidly evolving disease^5, 6^. The majority cases suffer from fatigue or pruritus in the early stage and are rarely suspected to have PBC. The diagnosis of PBC requires a combination of clinical features, auto-antibodies, abnormal liver biochemical patterns and liver histology; among them, the presence of serological AMAs and elevated alkaline phosphatase (ALP) are the most important and usually applied parameters^7^. However, liver chemical parameters and auto-antibodies are not pathogenic or required for the disease. AMA-negative PBC is difficult to diagnose because of the poor feasibility of liver biopsy in the suspected patients. Additionally, because many autoimmune diseases are associated with PBC, the differential diagnosis of PBC from other autoimmune liver diseases is very important. Therefore, improvement of the PBC diagnosis through the development of new methods to detect PBC accurately is important.

Metabolomics is a new “omic” discipline following genomics, transcriptomics and proteomics and is recognized as a promising technique for the evaluation of global metabolic changes^8^. By comparing metabolic profiles and their dynamic changes, characteristic changes including pathological and treatment changes can be elucidated. The current metabolomics methods include nuclear magnetic resonance (NMR), gas chromatography-mass spectrometry (GC/MS), and liquid chromatography-mass spectrometry (LC/MS). Recent reports have suggested that metabolic profiles may reveal novel characteristics of metabolic abnormalities of PBC and help improve the diagnosis^9–11^. Lv *et al*. revealed that gut microbiome was critical for the onset or development of PBC by interacting with metabolism and immunity^12^. Altamirano-Barrera *et al*. commented that high plasma concentrations of bile acids, in particular chenodeoxycholic acid, contribute to the induction of carcinogenesis via intrahepatic accumulation of bile acids^13^. Tang *et al*. showed that the levels of bile acids increased with the progression of PBC, while the levels of carnitines, such as propionyl carnitine and butyryl carnitine, decreased with the progression of PBC^14^. Moreover, cholestasis is associated with hyperlipidaemia, decreased fatty acid oxidation, increased glycolysis, and abnormal mitochondrial function^5, 15^. These profound metabolic changes hamper the renewal of mitochondria and contribute to oxidative damage^16^. As a consequence, an inflammatory cascade response is triggered and eventually evolves on set of fibrosis^5, 17^. Bell *et al*. identified four metabolic pathways that can be used as potential biomarkers to differentiate PBC from primary sclerosing cholangitis (PSC) patients, regarding protein/amino acid metabolism, lipid metabolism, oxidative stress/lipid peroxidation, and stress hormones^18^. Lian *et al*. and Wang *et al*. identified important differences of patients with PBC as compared to autoimmune hepatitis (AIH)^19, 20^. These metabolites could provide insight into metabolic pathways that contribute to the pathogenesis of PBC. However, the sensitivity and specificity of these biomarkers have been challenged and require further verification.

In the present study, we applied a high resolution ^1^H-NMR metabolomics approach to identify a distinctive signature in serum samples from PBC patients and to reveal the reprogramming of metabolic pathways in PBC. We established a diagnostic model including 4 metabolites (4-hydroxyproline, 3-hydroxyisovalerate, citraconate and pyruvate). Furthermore, we included 7 AIH patients, 9 HBC patients and 21 PBC patients in a validation dataset to validate the applicability and stability of the distinctive model. The results showed that the 4 novel metabolites were distinct for PBC, could differentiate PBC from HBC and AIH, and were not affected by ursodeoxycholic acid (UDCA) administration. These PBC-related distinctive metabolites could benefit from the development of novel categories of advanced disease biomarkers and assist in understanding the potential pathogenic mechanism.

## Results

### Baseline clinical characteristics of PBC patients

We collected 29 patients with PBC, 30 patients with HBC and 41 healthy controls in the training set. Among the patients with PBC, 23 were initially diagnosed, 6 were treated with UDCA (13–15 mg/kg/d) for more than one year and all had biochemical response. The baseline clinical characteristics of the PBC and HBC patients are shown in Table 1. The serum ALP, gamma-glutamyltransferase (γ-GT), total bile acid (TBA) and direct bilirubin (DBIL) levels significantly differed between the PBC and HBC patients (P < 0.05), but no significant differences were found in the age, liver ultrasonic score, and Child-Pugh score between the PBC and HBC patients (P > 0.05).

**Table 1:** Baseline clinical characteristics of the patients and controls in the training and validation sets.

	Training set (n = 100)			Validation set (n = 37)		
	PBC (n = 29)	HBC (n = 30)	Control (n = 41)	PBC (n = 21)	AIH (n = 7)	HBC (n = 9)
Age	61.00 ± 9.25	59.31 ± 10.71	59.48 ± 11.24	62.05 ± 10.00	60.29 ± 7.95	51.30 ± 11.52
Male/Female	5/24	5/25	10/31	5/16	0/7	0/9
ALT(IU/L)	73.25 ± 83.37	62.01 ± 104.25	/	47.30 ± 56.80	69.72 ± 59.05	30.78 ± 31.11
AST(IU/L)	81.91 ± 81.32	70.21 ± 118.25	/	69.35 ± 63.46	83.43 ± 59.10	34.67 ± 18.28
ALP(IU/L)	220.93 ± 125.65	122.76 ± 60.24^*^	/	225.05 ± 176.35	171.20 ± 131.82	87.13 ± 30.00
γ-GT(IU/L)	193.71 ± 175.19	77.91 ± 92.61^*^	/	159.46 ± 190.00	112.60 ± 123.60	25.90 ± 7.93
TBIL(µmol/L)	50.12 ± 87.28	30.86 ± 27.31	/	31.20 ± 27.34	51.02 ± 53.19	29.01 ± 7.44
DBIL(µmol/L)	22.32 ± 44.71	11.46 ± 14.40^*^	/	11.80 ± 16.42	24.34 ± 31.12	7.09 ± 3.43
GLB(g/L)	34.36 ± 5.66	31.15 ± 5.82	/	36.67 ± 9.38	38.18 ± 12.57	27.07 ± 4.76
ALB(g/L)	34.31 ± 5.43	31.03 ± 7.72	/	30.54 ± 6.32	29.85 ± 3.19	39.62 ± 8.23
TC(mmol/L)	4.94 ± 1.30	4.34 ± 3.32	/	4.20 ± 1.41	4.98 ± 3.90	3.52 ± 1.10
TBA(µmol/L)	79.91 ± 86.23	38.85 ± 44.20^*^	/	67.71 ± 51.49	82.27 ± 58.90	25.70 ± 32.06
CR(µmol/L)	59.18 ± 21.03	54.92 ± 19.84	/	63.95 ± 26.85	49.18 ± 11.77	50.58 ± 11.60
BUN(mmol/L)	5.05 ± 1.21	4.63 ± 1.63	/	5.71 ± 2.08	4.73 ± 1.09	4.69 ± 1.01
PT(S)	14.04 ± 2.07	15.79 ± 2.68	/	14.98 ± 2.14	15.80 ± 1.93	15.63 ± 2.23
INR(%)	1.09 ± 0.22	1.27 ± 0.30	/	1.18 ± 0.20	1.29 ± 0.21	1.27 ± 0.23
PLT(*10^9^/L)	109.67 ± 66.81	107.28 ± 100.57	/	95.01 ± 72.58	62.71 ± 14.40	114.78 ± 74.83
Liver ultrasonic score	3.52 ± 0.81	3.48 ± 0.87	/	3.65 ± 0.75	3.71 ± 0.49	3.00 ± 0.67
Child-Pugh score	6.43 ± 1.54	6.79 ± 1.57	/	6.95 ± 1.32	7.71 ± 1.50	5.60 ± 1.07

^*^P < 0.05 ALT: alanine aminotransferase, AST: aspartate aminotransferase, ALP: alkaline phosphatase, γ-GT: gamma-glutamyltranspeptidase, TBIL: total bilirubin, DBIL: direct bilirubin, GLB: globulin, ALB: albumin, TC: total cholesterol, TBA: total bile acid, CR: creatinine, BUN: blood urea nitrogen, PT: prothrombin time, INR: international normalized ratio, PLT: platelet count.

### Overall metabolomics analysis of serum samples

**Figure 1:**
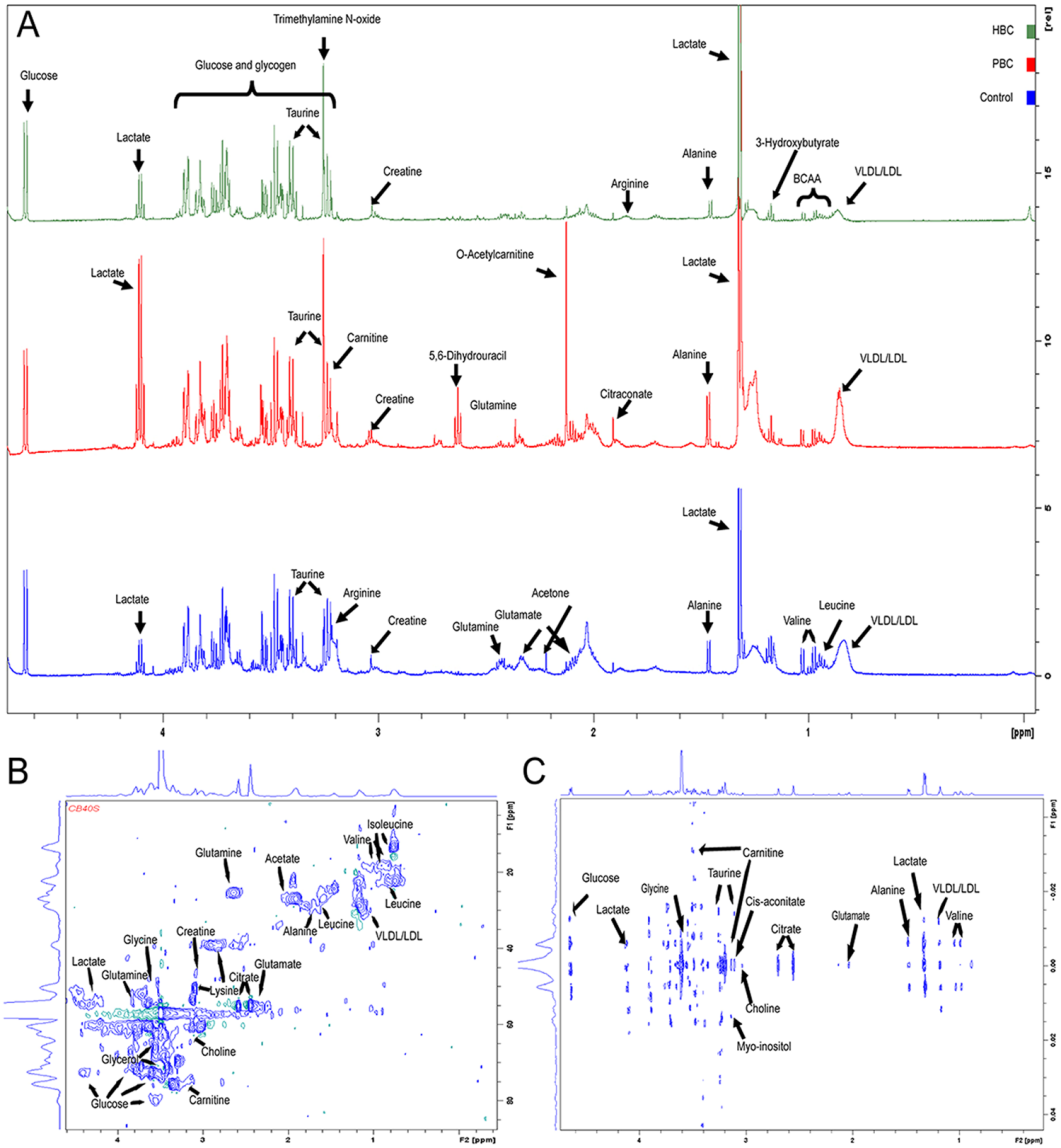
Typical NMR spectra of the three groups and the amplifying 2D spectra. (**A**) Metabolites were identified by representative 600 MHz NMR-based CPMG spectra. (**B** and **C**) 2D experiments of the HSQC and J-resolved spectra were used for the accurate identification and assignment of the metabolites in the highly crowded regions.

Representative NMR spectra with identified metabolites are shown in Fig. 1A. Because of overlap of the 1D spectrum, the splitting patterns and the coupling constants of each signal were not recognized. Thus, two-dimensional heteronuclear single quantum coherence (2D-HSQC) and J-resolved spectra experiments were used to accurately identify and assign the metabolites in the highly crowded regions (Fig. 1B and C). The serum spectra contained high-intensity signals from the small molecules lactate, alanine, creatine, taurine, glycine, and glucose. Although the overall spectrum signals were quite similar for the three groups, seemingly specific signals were identified in each group (Supplementary Material 1). Statistical evaluation by principal component analysis (PCA) showed clear separation. The wider spread of the samples in the PCA was caused by different levels of metabolic manifestation within-subject and/or by date of subsequent sampling. Orthogonal partial least squares discriminant analysis (OPLS-DA) was applied to exclude possible confounding variables not related to the group differences and to evaluate the statistical meaning of those signals. This result was highly encouraging because the discrimination model could differentiate the three groups without any overlap in one orthogonal component. However, the models had quite high cross-validated predictability and goodness-of-fit values, with a Q^2^(Y) of 0.950, an R^2^(Y) of 0.987, and a cumulative R^2^(X) of 0.852, indicating reliable differentiation between the groups.

### Serum metabolites related to PBC

**Figure 2:**
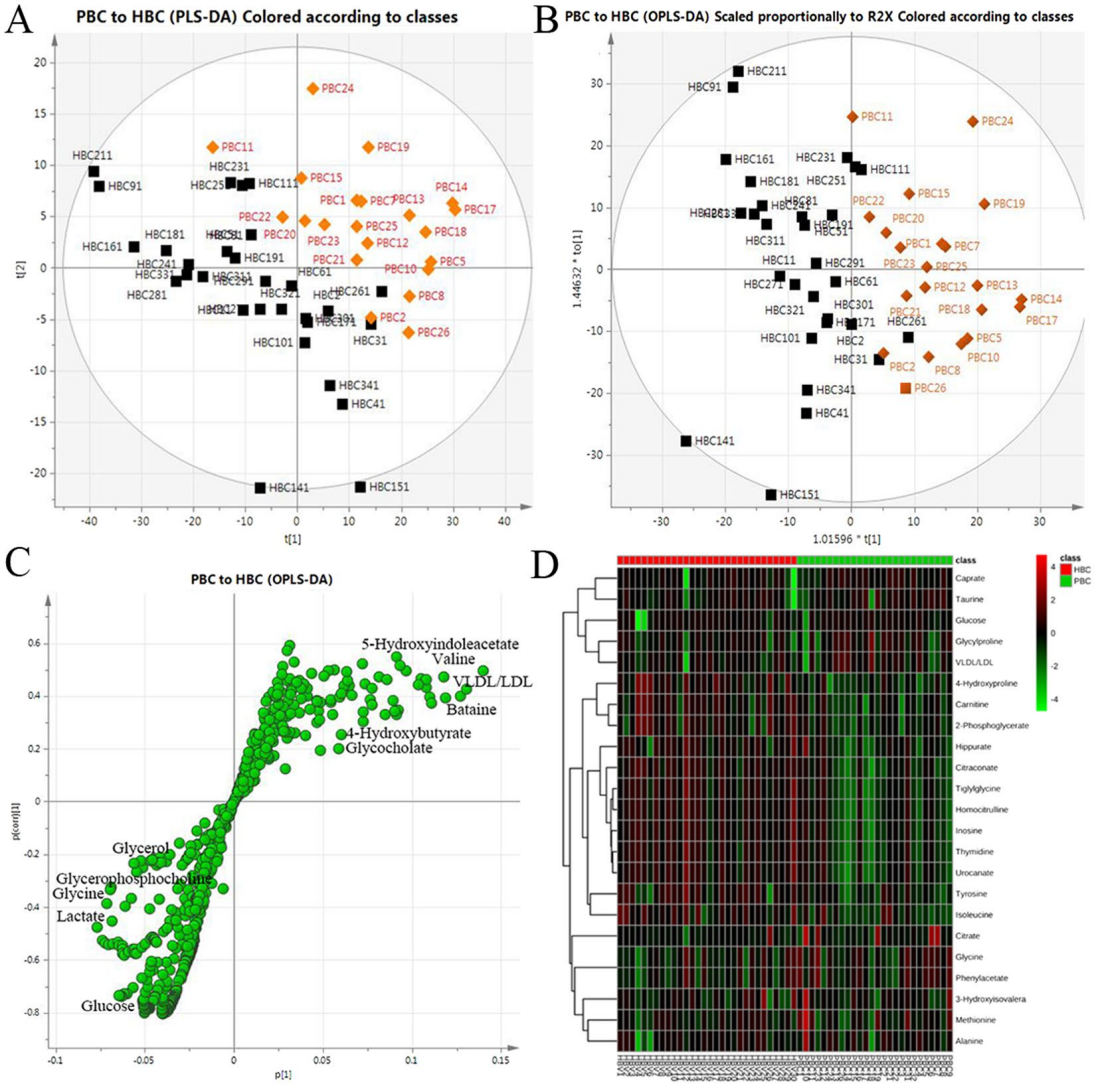
The serum PCA score plot (**A**), OPLS-DA (**B**), S-plot (**C**) and heat map (**D**) used to classify the PBC and HBC patients obtained a satisfactory validation. A total of 25 metabolites were analyzed, and the results indicated that the PBC and HBC patients could be separated based on these metabolites.

A pairwise comparison was performed between the PBC and HBC patients to discover the distinct potential biomarkers responsible for PBC among the thousands of variables. The PCA model for the classification of the two groups obtained satisfactory validation (Fig. 2A). The OPLS-DA model was used to elucidate the most reliable class-discriminating variables that were highly diagnostic for group separation. One principal component (PC) could differentiate the two groups (Fig. 2B). The visualized S-plot (Fig. 2C) was applied with p1 and p1 (corr) set as >±0.05.Together with the variable importance in the projection (VIP) from the above OPLS-DA model, a total of 25 metabolites with a VIP >1 were selected, including increasing levels of very low-density lipoprotein/low-density lipoprotein (VLDL/LDL), taurine, glycine, phenylacetate, citrate, caprate, glycylproline, glucose, 3-hydroxyisovalerate, methionine, and alanine and decreasing levels of 4-hydroxyproline, carnitine, 2-phosphoglycerate, citraconate, tyrosine, 3-hydroxyisobutyrate, inosine, thymidine, ornithine, tiglylglycine, urocanate, hippurate, n-acetylcysteine, and isoleucine. Then, the metabolites were analyzed using a heat map. The results indicated that the two groups could be separated based on these metabolites (Fig. 2D).

**Figure 3:**
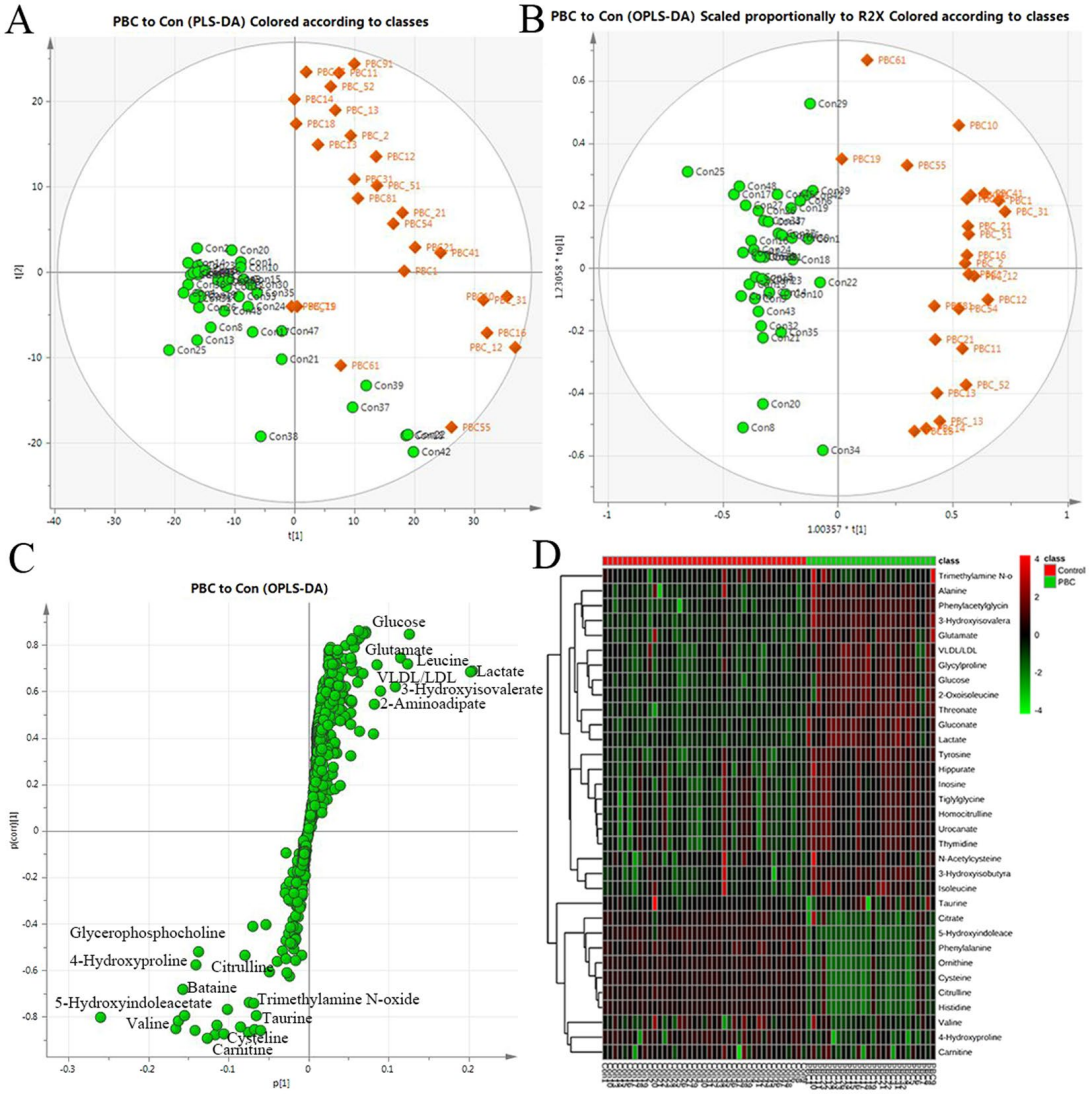
The serum PCA score plot (**A**), OPLS-DA (**B**), S-plot (**C**) and heat map (**D**) used to classify the PBC patients and healthy controls gained a clear separation and identified 33 metabolites. These metabolites could distinguish between the PBC patients and healthy controls.

To lower the risk of false positives in the metabolite selection procedure, another two pairwise comparisons were performed. The score plot from the PCA model of the pairwise comparison between the PBC and healthy control groups also showed a clear separation (Fig. 3A). The model for the classification of the two groups obtained a satisfactory validation with an R^2^Y (cum) of 0.834 and a Q^2^ (cum) of 0.759 when the OPLS-DA model was used (Fig. 3B). The visualized S-plot (Fig. 3C) and the variables with high VIPs indicated 33 metabolites, including increasing levels of phenylacetylglycine, 2-oxoisoleucine, threonate, 4-hydroxyproline, gluconate, glucose, glycylproline, 3-hydroxyisovalerate, 2-aminoadipate, glycerol, glutamate, tyrosine, alanine, 3-hydroxyisobutyrate, homocitrulline, isoleucine, hippurate, urocanate, thymidine, inosine, and tiglylglycine and decreasing levels of 5-hydroxyindoleacetate, valine, phenylalanine, citrulline, histidine, cysteine, citrate, ornithine, taurine, carnitine, trimethylamine n-oxide, and n-acetylcysteine. The heat map indicated that these metabolites could distinguish the PBC patients from the healthy controls (Fig. 3D).

**Figure 4:**
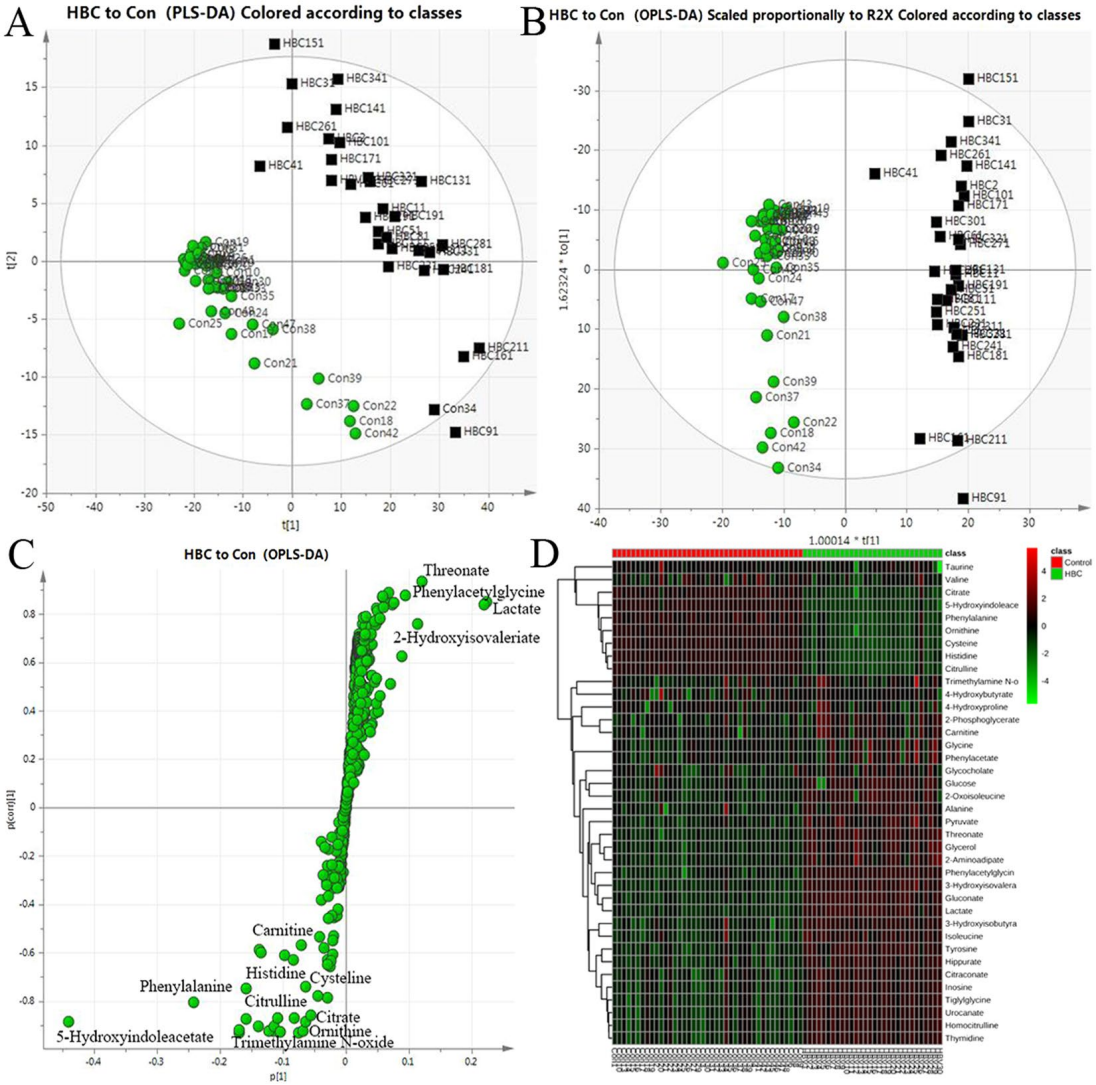
The serum PCA score plot (**A**), OPLS-DA (**B**), S-plot (**C**) and heat map (**D**) used to distinguish between the HBC patients and healthy control groups. A total of 40 metabolites were identified that could distinguish between the HBC patients and healthy controls.

The PCA score plot (Fig. 4A) showed a clearly different distribution of the HBC patients and healthy controls from the two primary PCs. The OPLS-DA model was used to recognize the metabolites. The score plot (Fig.4B) and S-plot (Fig. 4C) of the OPLS-DA model from the pairwise comparison between the HBC patients and healthy controls included increasing levels of glucose, gluconate, glycerol, pyruvate, 3-hydroxyisovalerate, 2-oxoisoleucine, 3-hydroxyisobutyrate, alanine, 2-phosphoglycerate, hippurate, tyrosine, tiglylglycine, urocanate, homocitrulline, thymidine, inosine, citraconate, isoleucine, carnitine, 2-aminoadipate, threonate, phenylacetylglycine, glutamate, and 4-hydroxyproline and decreasing levels of taurine, citrulline, 4-hydroxybutyrate, 5-hydroxyindoleacetate, valine, glycine, trimethylamine n-oxide, phenylalanine, cysteine, histidine, ornithine, citrate, glycocholate, methionine, n-acetylcysteine, and phenylacetate. These metabolites revealed that the HBC patients possessed a highly unique metabolic phenotype characterized by glycolysis, the mitochondrial citric cycle acid, and choline and fatty acid metabolism reprogramming. To filter the different metabolites resulting from HBC, the pairwise comparison screened potential biomarkers related to HBC. The heat map results also indicated that these metabolites could distinguish HBC patients from the healthy controls (Fig. 4D).

UDCA is the only drug approved by the Food and Drug Administration (FDA) for the treatment of PBC, but approximately 30 to 40% of patients may not achieve a biochemical response with UDCA treatment^21^. Because the pharmacodynamic biomarkers are important, we analyzed whether UDCA caused metabolic changes in the PBC patients. A total of 6 of the 29 PBC patients in the training set were treated with UDCA for more than 1 year. Their serum samples were analyzed for metabolic profiling. Interestingly, the result revealed that there is no significant difference between the UDCA treated and naїve PBC patients on the above serum metabolic profiles. This result suggested that PBC patients could have some metabolic characteristics associated with the intrinsic properties of the disease.

### Metabolite profile and pathways related to PBC

**Figure 5:**
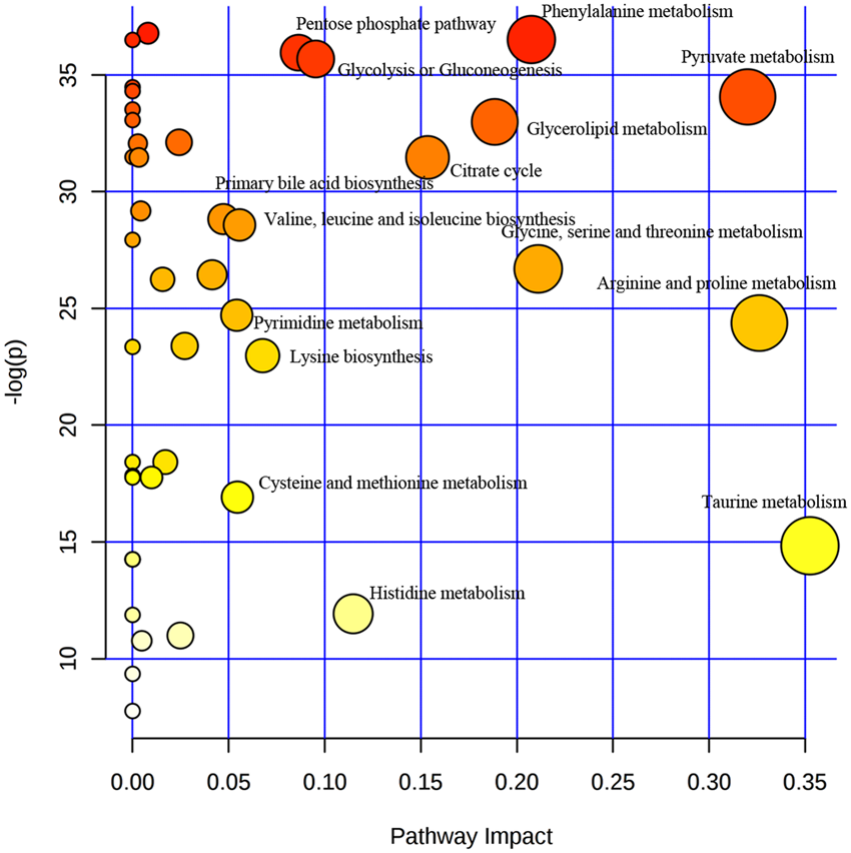
Metabolic pathway analysis. The χ-axis represents the pathway impact, and the *y-*axis represents the −log (*p*). The pathway analysis result showed the metabolic network reprogramming of PBC with detailed impact.

The normalized concentrations of the examined metabolites in the three groups are shown in Fig. 5. All of the attributed metabolites were analyzed using Metaboanalyst 3.0. The pathway analysis results showed the metabolic network reprogramming of PBC with detailed impact. The most influenced metabolic pathway was set as a pathway impact >0.05 and −log (*p*) >10. The biological pathway analysis revealed fifteen metabolic pathways, including taurine metabolism, arginine and proline metabolism, pyruvate and citrate metabolism, glycine, serine and threonine metabolism, phenylalanine metabolism, glycerolipid metabolism, butanoate metabolism, glycolysis or gluconeogenesis, pentose phosphate pathway, lysine biosynthesis, valine, leucine and isoleucine biosynthesis, cysteine and methionine metabolism, pyrimidine metabolism, and primary bile acid biosynthesis (Supplementary Material 2).

### Selection of distinctive metabolites

**Figure 6:**
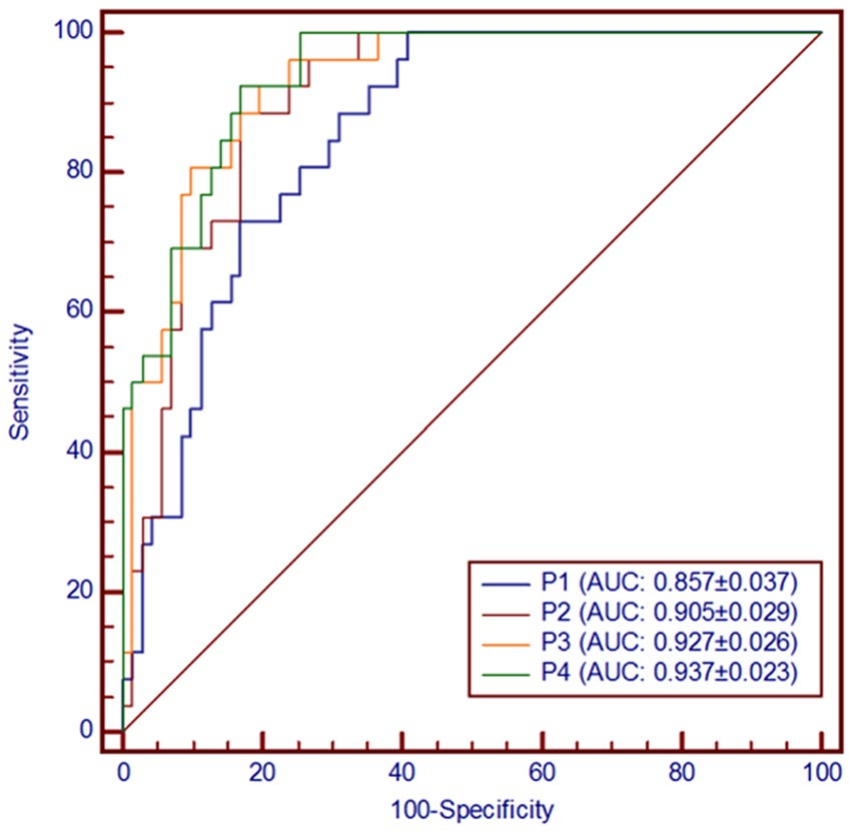
The ROC analysis results from the four diagnostic models calculated from the logistic regression analysis. The performance of each biomarker model was evaluated by the area under the ROC curve (AUC) and the determination of sensitivity and specificity at the optimal cut-off point defined by the minimum distance to the top-left corner. The optimized model was the P4 model with an AUC of 0.937 (95% CI: 0.868–0.976).

Establishing a diagnostic model to predict the presence of PBC was difficult because of the distinct metabolic profile of PBC consisting of the fifteen altered metabolic pathways and 45 corresponding metabolites. To improve the prediction of PBC, a combination of more than one discriminatory metabolite was developed using the training set through logistic regression analysis via forward stepwise selection. The sensitivity and specificity were summarized using the area under the curve (AUC). The performance of the designed model was evaluated by the AUC in the training set. Notably, 4-hydroxyproline, 3-hydroxyisovalerate, citraconate and pyruvate were present in the combined model. None of the other metabolites were included in the model, likely because of colinearity in the information provided by these compounds. Thus, the four metabolites contributed to the combined model. We also constructed receiver operating characteristic (ROC) curves for the four individual metabolites independently. Table 2 shows that the combination of metabolites was a better discriminator (AUC > 90% in the training set) than each metabolite individually (AUC < 90%), which reinforced the improved capacity of biomarker patterns to distinguish between different groups. The performance of the distinctive model with the highest AUC was equated as (*p*) = −12.22–3.46* log (4-hydroxyproline) + 6.62* log (3-hydroxyisovalerate) − 2.44* log (citraconate) − 3.80* log (pyruvate). The corresponding ROC curve had an AUC of 0.937 (95% confidence interval (CI): 0.868–0.976) with a Youden index J of 0.754 (Fig. 6). Thus, the distinctive signature with the four metabolites achieved the highest AUC value and significantly increased the diagnostic performance of PBC. The corresponding sensitivity was 69.23%, and the specificity was 92.69% (Supplementary Materials 1 and 3).

**Table 2:** Diagnostic models from the logistic regression and the ROC analysis results.

	Diagnosis models	AUC	SE^a^	95% CI^b^	YouDen index J	Sensitivity	Specificity
P1	(*p1*) = −12.17–3.79*log(4-hydroxyproline)	0.857	0.0373	0.771 to 0.920	0.592	57.69	88.73
P2	(*p2*) = 5.27–2.97* log(4-hydroxyproline) +5.35*log(3-hydroxyisovalerate)	0.905	0.0296	0.829 to 0.955	0.716	69.23	91.55
P3	(*p3*) = −4.02–3.72* log(4-hydroxyproline) +5.81*log(3-hydroxyisovalerate) −2.36*log(citraconate)	0.927	0.0257	0.856 to 0.970	0.725	80.77	90.14
P4	(*p4*) = −12.22–3.46* log(4-hydroxyproline) +6.62*log(3-hydroxyisovalerate) −2.44*log(citraconate) −3.80*log(pyruvate)	0.937	0.0227	0.868 to 0.976	0.754	69.23	92.69

^a^Standard Error; ^b^Confidence interval.

### Validation of the distinctive model in a different set

To validate the applicability and stability of the distinctive model, we collected and analyzed serum samples from 21 PBC, 7 AIH and 9 HBC patients in an experiment using a different data set. The clinical baseline characteristics of the three patient groups are listed in Table 1. The AUC was 0.890 (95% CI: 0.743–0.969) with a Youden index J of 0.7024 (Supplementary Material 4) in the validation set, and the corresponding sensitivity and specificity were 95.24 and 75.00%, respectively. Thus, the distinctive signature of the four metabolites significantly increased the diagnostic performance of PBC.

## Discussion

PBC has very diverse clinical and laboratory manifestations. Thus, the diagnosis of PBC is challenging, particularly its early diagnosis and differential diagnosis from other autoimmune liver diseases. Therefore, an early and accurate diagnosis of the disease is important to lower liver-related morbidity and mortality. In the current study, we used high resolution ^1^H-NMR spectroscopy to identify a distinctive PBC signature. First, we detected 45 distinct metabolites from the comparative analysis and 15 metabolic pathways that exhibited reprogramming. To select and validate marker metabolites, a logistic regression and ROC analysis were used to establish a diagnostic model with 4 metabolites (4-hydroxyproline, 3-hydroxyisovalerate, citraconate and pyruvate). The AUC of the optimized model was 0.937 (95% CI: 0.868–0.976), which indicated satisfactory sensitivity and specificity. Then, we performed an independent confirmatory experiment including 21 patients with PBC, 7 AIH patients and 9 patients with HBC and found that the combination of the above 4 metabolites effectively differentiated PBC from AIH and HBC. The sensitivity and specificity of distinguishing each sample were 95.24 and 75.00%, respectively, which indicated that the model had a high clinical value for the diagnosis of PBC.

4-Hydroxyproline is a specific amino acid in collagen protein that plays a key role in collagen stability and reflects the collagen content or fibrosis stage. Moreover, an increased serum or urinary hydroxyproline level is associated with depression^22^ and fatigue^23^. 3-Hydroxyisovalerate (3-HIVA) is produced from 3- hydroxy-3- methylglutary- CoA, which is a side product of increased ketogenesis^24^. A high 3-HIVA concentration occurs in ketoacidotic and isovaleric acidemia patients^25^. Citraconate is an isomeric carboxylic acid that is obtained from citrate, and recent studies have revealed that it is a competitive inhibitor of fumarate reduction^26^. Pyruvate is an intermediate compound in the metabolism of carbohydrates, proteins, and fats^27^, which could be regulated by pyruvate dehydrogenase. While AMA is directed against the lipoyl domain of the E2 subunit of pyruvate dehydrogenase^27, 28^, the E2 subunit can be chemically modified and triggers the breakage of tolerance^29, 30^, which will contribute to the pathogenesis of PBC^31^. These findings partially indicated that pyruvate metabolism specificities reflected aspects of the PBC induction phase. Therefore, the 4 metabolites have significance for PBC and are good biomarkers for the PBC diagnosis.

In addition to the forty-five metabolites, the metabolic pathway analysis revealed fifteen reprogramming pathways, mainly including taurine metabolism, pyruvate and citrate metabolism, glycine, serine and threonine metabolism, glycerolipid metabolism, glycolysis or gluconeogenesis, and the pentose phosphate pathway. These changed pathways embody the potential pathological mechanisms of PBC and warrant further in-depth research.

UDCA is effective for PBC treatment and can delay disease progression in most PBC patients. However, UDCA influences the bile acid assay in serum because UDCA itself is a type of bile acid. In our study, we found that UDCA treatment did not cause significant metabolite changes compared to the UDCA-naїve patients. This finding indicates that the metabolic profile does not reflect the therapeutic response or function as a metabolic marker, suggesting that the established metabolic diagnostic model can specifically distinguish PBC and is not associated with conventional therapy. We will expand the number of samples and compare metabolic differences between UDCA responders and non-responders in future studies.

Nevertheless, we could not analyze the bile acids in the present study for the constraint of the NMR platform. However, abnormalities in lipoprotein and bile acid metabolism have been well described^14, 32^. We found that hyperlipidemia and glucose metabolic disorders were common in PBC patients. Investigations of the overall metabolic changes have been quite limited, but the metabolic profile may reveal a distinctive PBC signature. ^1^H-NMR-based metabolomics can provide non-destructive, high-throughput metabolic profiles with minimal sample preparation. The technique recognizes metabolites by measuring the intrinsic properties of each proton and can distinguish low molecular weight metabolites through chemical shift, splitting patterns and coupling constants. ^1^H-NMR-based metabolomics is an unbiased method to distinguish metabolites and is extensively used in metabolic analyses. In the present study, high resolution ^1^H-NMR-based metabolomics was employed to reveal the metabolic profiles and global metabolites. A total of forty-five metabolites were changed in the three groups. Thus, the high-resolution NMR-based metabolomics approach was feasible for the present study.

In conclusion, forty-five distinguishable metabolites were identified and fifteen metabolic pathways were reprogrammed. Logistic regression and ROC analysis were used to establish a diagnostic model with the equated (p) = −12.22–3.46* log (4-hydroxyproline) + 6.62* log (3-hydroxyisovalerate) − 2.44*log (citraconate) − 3.80*log (pyruvate). The AUC of the optimized model was 0.937 (95% CI: 0.868–0.976) in the training set and 0.890 (95% CI: 0.743–0.969) in the validation set. Therefore, the distinctive signature of the four metabolites significantly increased the diagnostic performance of PBC.

## Materials and Methods

### Patient selection

Ethical approval was obtained from the Health Research Ethics Board at Shuguang Hospital Affiliated with Shanghai University of Traditional Chinese Medicine, and all methods were performed in accordance with the relevant guidelines and regulations. A total of 29 patients with PBC and 30 cases of HBC were recruited into the training set in July 2015 to June 2016 from the Department of Cirrhosis. A total of 41 healthy controls were randomly selected from our hospital health examination center. The validation set included an additional 21 PBC, 7 AIH and 9 HBC patients collected in May 2016 to November 2016. The patients with AIH were diagnosed according to the revised scoring system proposed by the International Autoimmune Hepatitis Group (IAIHG) in 1999^33^. All participants provided written informed consent. Information regarding the age, gender, the duration of illness, laboratory indices, liver ultrasonic score, Child-Pugh score and previous diseases was collected. For ethical reasons, the patients continued to take their prescribed medications at the time of serum sampling. The full diagnostic assessment was based on all available sources of information, including patient interviews, case records, and if possible, interviews with the next of kin. To reduce inter-rater bias, a diagnosis decision based on all information available at admission was made by a consensus panel of board specialists in PBC. The general criteria for inclusion were as follows: at least 18 years of age and meeting the diagnostic criteria for PBC. The exclusion criteria were as follows: chemotherapy closest to the sample acquirement; cardiac, kidney, or liver failure; acquired immunodeficiency syndrome; inflammatory bowel disease; and systemic infection.

### Sample collection

Morning fasting blood was collected in a vacuum tube and kept at room temperature for 30 min to allow clotting. The clotted blood samples were centrifuged at 3000 × *g* at 4 °C for 20 min to eliminate the supernatant serum and then quickly stored at −80 °C prior to the execution of the ^1^H-NMR experiment. To obtain robust and reproducible data, all samples were prepared according to the standard protocol^34^. Briefly, the samples were thawed at 4 °C for 2 h and then left at room temperature for 1 h. Then, 300 μL of thawed sample was mixed with 300 μL of phosphate buffer (pH 7.4) containing 10% D_2_O. After vortex mixing and centrifugation, 550 μL of the solution was transferred into 5 mm NMR tubes and kept at 4 °C prior to the NMR analysis.

### ^1^H-NMR spectroscopy measurement protocols

All spectra were recorded using a Bruker AMX-600 NMR spectrometer operated at a 600.13 MHz ^1^H resonance frequency (Bruker Biospin, Rheinstetten, Germany). Quality control tests were performed at the beginning of each measurement day. A representative sample was used for NMR probe tuning and matching, determination of the transmitter offset value for water pulse presaturation and 90 pulse adjustments. Each sample was locked and shimmed automatically; the receiver gain was set to 90.5 and the temperature to 310 K for all experiments. The CPMG (Carr–Purcell–Meiboom–Gill) pulse sequence was employed to obtain the low molecular signal with the equation-delay-90°-(t-180°-t) n - acquisition, where the relaxation delay was 2 s and t was the spin-echo delay of 400 μs. All signals were zero-filled to 32 k prior to Fourier transformation (FT). All spectra were acquired and performed using the TopSpin software package version 3.0 (Bruker Biospin, Rheinstetten, Germany).

### Metabolite identification and chemical signal assignment

The Chenomx NMR Suite software version 8.2 (Chenomx, Inc., Alberta, Canada) was used to identify metabolites from the ^1^H-NMR spectra of biological samples. Because of the overlap of signals from the ^1^H-NMR spectra, 2D-HSQC and J-Resolved spectra were employed for the metabolite identification and signal assignment. The 2D-HSQC spectrum provided excellent spectral dispersion along the indirect 13C dimension and allowed the separation of many of the peaks that overlapped in the 1D NMR spectrums. The J-Resolved spectra were acquired using 16 transients per increment for a total of 32 increments that were collected into 16 k data points with spectral widths of 6 kHz along the direct chemical shift axis and 50 Hz along the spin–spin coupling axis. Prior to Fourier transformation, the datasets were zero-filled to 128 points in the spin–spin coupling axis and 32,768 points in the chemical shift axis.

### Data reduction and metabolomics analysis

Phase correction and baseline correction were carefully performed, and the ^1^H chemical shifts referred to the methyl doublet signal of lactate (*δ* 1.33). The corrected NMR spectra corresponding to the chemical shift range of *δ* 0.2–10.0 were imported into AMIX 3.9.5 (Bruker Biospin, Rheinstetten, Germany), and all of the spectra were reduced into integral regions with equal lengths of 0.005 ppm. Regions of *δ* 4.7–5.1 that contained the resonance from residual water were set to zero. To reduce the concentration differences between samples, the data were normalized to the total spectral area (100%).The datasets were analyzed by pattern recognition methods using the software packages Simca-P, version 11.5 (UmetricsAB, Umea, Sweden), and MetaboAnalyst 3.0 (www.metaboanalyst.ca). To make the skewed distributions more symmetric, log transformations were used for nonlinear conversions of the data. To explain the maximum variation between samples, a PCA bilinear decomposition method was used to view the clusters within the multivariate data. To eliminate the effect of inter-subject variability among the participants and identify endogenous metabolites that significantly contributed to the classification, OPLS-DA was applied to remove linear combinations of variable X orthogonal to the Y vector. Cumulative R^2^(Y) and Q^2^(Y) values close to 1 indicated an excellent model. The specific metabolites between classes were interpreted using VIP and the correlation coefficient.

### Metabolic network analysis

To identify the most relevant metabolic pathways involved in PBC, a metabolomics pathway analysis was employed to perform the pathway enrichment and pathway topology analyze. The metabolites with VIP scores >1.0 in the partial least squares (PLS) or p1 >±0.05 in the S-Plot were examined and selected for their discrimination power by multiple statistical criteria. A global ANOVA was used to analyze the concentration values and identify subtle changes, and a relative betweenness centrality was used for the metabolite importance measurement. The changed metabolites in the network node were selected because they would trigger a more severe impact on the pathway than changes that occurred in marginal or relatively isolated positions.

### Selection of biomarker candidates

We designed a prediction model and constructed ROC curves to evaluate the accuracy of the model. First, the data set was split into training and validation. Then, a forward stepwise logistic regression model was constructed on the training sample set to design the best metabolite combination. ROC curves were used to evaluate the accuracy of this model in the training and validation sets and to evaluate the combined model following the DeLong method. ROC curve analyses were performed for the designed model. The global performance of each biomarker model was evaluated using the AUC and the determination of sensitivity and specificity at the optimal cut-off point defined by the minimum distance to the top-left corner.

### Statistics

Data entry and analysis were performed using MedClac software version 13.0.6.0 (Broekstraat, Mariakerke, Belgium). After data collection, the data were checked manually for completeness and inconsistencies. The attribute data were expressed as the counts and analyzed using the non-parametric crosstab method. The measurement data are expressed as the mean ± SD (standard deviation). The differences between the two groups were analyzed using Student’s two-sided *t*-test, and differences involving more than two groups were analyzed using a one-way ANOVA followed by Tukey’s posthoc test. The level of significance was set at p < 0.05.

## Electronic supplementary material

Supplementary Material 1

Supplementary Material 2

Supplementary Material 3

Supplementary Material 4
