# Supplementary material for: Serum Metabolomics Analysis Reveals a Distinct Metabolic Profile of Patients with Primary Biliary Cholangitis
Source: Sci Rep. 2017 Apr 11;7:784. doi: 10.1038/s41598-017-00944-9 (PMC5429753; doi:10.1038/s41598-017-00944-9)
Supplement: Supplementary file 2 — Supplementary Material 2 [file 41598_2017_944_MOESM2_ESM.pdf]

Serum Metabolomics Analysis Reveals a Distinct Metabolic Profile of Patients with Primary Biliary Cholangitis

Juan Hao[1]. Tao Yang[1,2]. Yang Zhou[1], Guo-Yuan Gao[1,3], Feng Xing[1], Yuan Peng[1], Yan-Yan Tao[1], and Cheng-Hai Liu[1,4,5]

1 Institute of Liver Diseases, Shuguang Hospital Affiliated to Shanghai University of Traditional Chinese Medicine, 528 Zhangheng Road, Shanghai 201203, China

2 Institute of Cardiovascular Disease, Shuguang Hospital Affiliated to Shanghai University of Traditional Chinese Medicine, Shanghai 201203, China

3 School of Pharmacy, East China University of Science and Technology, Shanghai 200237, China

4 E-Institute of Traditional Chinese Medicine Internal Medicine, Shanghai Municipal Education Commission, 1200 Cailun Road, Shanghai 201203, China

5 Shanghai Key Laboratory of Traditional Chinese Clinical Medicine, Shanghai 201203, China

Correspondence author:

Cheng-Hai Liu, Institute of Liver Diseases, Shuguang Hospital Affiliated to Shanghai University of Traditional Chinese Medicine, 528 Zhangheng Road, Shanghai 201203, China; E-mail: chenghailiu@hotmail.com

Supplementary material

Supplementary material 2: Pathway analysis with MetaboAnalyst 3.0

Metabolomic Data Analysis with MetaboAnalyst 3.0

User ID: guest3778016179802062138

August 8, 2016

1 Background

The Pathway Analysis module combines results from powerful pathway enrichment analysis with the pathway topology analysis to help researchers identify the most relevant pathways involved in the conditions under study.

There are many commercial pathway analysis software tools, such as Pathway Studio, MetaCore, or Ingenuity Pathway Analysis (IPA), etc. Compared to them, the pathway analysis module was specially developed for metabolomics studies. It uses the high-quality KEGG metabolic pathways as the backend knowledgebase. It integrates many well-established (i.e. univariate analysis, over-representation analysis) methods, as well as novel algorithms and concepts (i.e. Global Test, GlobalAncova, network topology analysis) into pathway analysis. Another feature is a Google-Map style interactive visualization system to deliver the analysis results in an intuitive manner.

2 Data Input

Pathway Analysis accepts either a list of compound labels (common names, HMDB IDs or KEGG IDs) with one compound per row, or a compound concentration table with samples in rows and compounds in columns. The second column must be phenotype labels (binary, multi-group, or continuous). The table is uploaded as comma separated values (.csv).

3 Compound Name Matching

The first step is to standardize the compound labels used in user uploaded data. This is a necessary step since these compounds will be subsequently compared with compounds contained in the pathway library. There are three outcomes from the step - exact match, approximate match (for common names only), and no match. Users should click the textbfView button from the approximate matched results to manually select the correct one. Compounds without match will be excluded from the subsequently pathway analysis.

Table 1 shows the conversion results. Note: *1* indicates exact match, *2* indicates approximate match, and *0* indicates no match. A text file contain the result can be found the downloaded file *name_map.csv*

Table 1: Result from Compound Name Mapping

	Query	Match	HMDB	PubChem	KEGG	Comment
1	Glucose	D-Glucose	HMDB00122	5793	C00031	1
2	4-Hydroxyproline	4-Hydroxyproline	HMDB00725	5810	C01157	1
3	Gluconate	Gluconic acid	HMDB00625	10690	C00257	1
4	Glycerol	Glycerol	HMDB00131	753	C00116	1
5	2-Aminoadipate	Aminoadipic acid	HMDB00510	469	C00956	1
6	Threonate	Threonic acid	HMDB00943	151152	C01620	1
7	Phenylacetylglycine	Phenylacetylglycine	HMDB00821	68144	C05598	1
8	4-Hydroxybutyrate	4-Hydroxybutyric acid	HMDB00710	10413	C00989	1
9	5-Hydroxyindoleacetate	5-Hydroxyindoleacetic acid	HMDB00763	1826	C05635	1

10	Valine	L-Valine	HMDB00883	6287	C00183	1
11	Glycine	Glycine	HMDB00123	750	C00037	1
12	Trimethylamine N-oxide	Trimethylamine N-oxide	HMDB00925	1145	C01104	1
13	Betaine	Betaine	HMDB00043	247	C00719	1
14	Glycerophosphocholine	Glycerophosphocholine	HMDB00086	71920	C00670	1
15	Taurine	Taurine	HMDB00251	1123	C00245	1
16	Phenylalanine	L-Phenylalanine	HMDB00159	6140	C00079	1
17	Citrulline	Citrulline	HMDB00904	9750	C00327	1
18	Cysteine	Cysteine	METPA0075		C00736	1
19	Histidine	L-Histidine	HMDB00177	6274	C00135	1
20	Ornithine	Ornithine	HMDB00214	6262	C00077	1
21	Citrate	Citric acid	HMDB00094	311	C00158	1
22	Pyruvate	Pyruvic acid	HMDB00243	1060	C00022	1
23	3-Hydroxyisovalerate	3-Hydroxyisovaleric acid	HMDB00754	69362		1
24	Glutamate	D-Glutamic acid	HMDB03339	23327	C00217	1
25	Lactate	L-Lactic acid	HMDB00190	107689	C00186	1
26	Fatty acid	Fatty acid	METPA0012		C00162	1
27	Leucine	L-Leucine	HMDB00687	6106	C00123	1
28	Glycocholate	Glycocholic acid	HMDB00138	23617285	C01921	1
29	2-Oxoisoleucine	3-Methyl-2-oxovaleric acid	HMDB00491	47	C03465	1
30	Caprate	Capric acid	HMDB00511	2969	C01571	1
31	3-Hydroxyisobutyrate	(S)-3-Hydroxyisobutyric acid	HMDB00023	440873	C06001	1
32	Alanine	Alanine	METPA0179		C01401	1
33	Glycylproline	Glycylproline	HMDB00721	79101		1
34	Methionine	L-Methionine	HMDB00696	6137	C00073	1
35	N-Acetylcysteine	Acetylcysteine	HMDB01890	12035	C06809	1
36	Phenylacetate	Phenylacetic acid	HMDB00209	999	C07086	1
37	2-Phosphoglycerate	2-Phosphoglyceric acid	HMDB00362	59		1
38	Carnitine	Carnitine	METPA0048		C00487	1
39	Hippurate	Hippuric acid	HMDB00714	464	C01586	1
40	Tyrosine	L-Tyrosine	HMDB00158	6057	C00082	1
41	Tiglylglycine	Tiglylglycine	HMDB00959	6441567		1
42	Urocanate	Urocanic acid	HMDB00301	736715	C00785	1
43	Homocitrulline	Homocitrulline	HMDB00679	65072	C02427	1
44	Thymidine	Thymidine	HMDB00273	5789	C00214	1
45	Inosine	Inosine	HMDB00195	6021	C00294	1
46	Citraconate	Citraconic acid	HMDB00634	643798	C02226	1
47	Isoleucine	(+/-)-erythro-Isoleucine	HMDB33923	791	C16434	1

4 Pathway Analysis

In this step, users are asked to select a pathway library, as well as specify the algorithms for pathway enrichment analysis and pathway topology analysis.

4.1 Pathway Library

There are 15 pathway libraries currently supported, with a total of 1173 pathways :

- Homo sapiens (human) [80]
- Mus musculus (mouse) [82]
- Rattus norvegicus (rat) [81]
- Bos taurus (cow) [81]
- Danio rerio (zebrafish) [81]
- Drosophila melanogaster (fruit fly) [79]
- Caenorhabditis elegans (nematode) [78]
- Saccharomyces cerevisiae (yeast) [65]
- Oryza sativa japonica (Japanese rice) [83]
- Arabidopsis thaliana (thale cress) [87]
- Escherichia coli K-12 MG1655 [87]
- Bacillus subtilis [80]
- Pseudomonas putida KT2440 [89]
- Staphylococcus aureus N315 (MRSA/VSSA)[73]
- Thermotoga maritima [57]

Your selected pathway library code is **hsa** (KEGG organisms abbreviation).

4.2 Pathway Enrichment Analysis

Pathway enrichment analysis usually refers to quantitative enrichment analysis directly using the compound concentration values, as compared to compound lists used by over-representation analysis. As a result, it is more sensitive and has the potentail to identify **subtle but consistent** changes among compounds involved in the same biological pathway.

Many procedures have been developed in the last decade for quantitative enrichment analysis, the most famous being the Gene Set Enrichment Analysis. Many new and improved methods have been implemented ever since. The enrichment analysis is based on GlobalTest and GlobalAncova. Both methods support enrichment analysis with binary, multi-group, as well as continuous phenotypes. The p values can be approximated based on the asymptotic distribution without using permutations which is computationally very intensive and is not suitable for web applications. Please note, when sample sizes are small, the approximated p values may be slightly less accurate compared to p values obtained by permutation-based method (for details, please refer to the paper by Goeman, J.J. et al. [1] and by

[1] Jelle J. Goeman and Peter Buhlmann. *Analyzing gene expression data in terms of gene sets: methodological issues*, Bioinformatics 2007 23(8):980-987

Hummel, M. et al. [2]) However, since our focus is to identify the most relevant pathways within the pathways in the library, we are more interested in the rank of the pathway, not its absolote p-value. Therefore, this disadvantage may be tolerated.

The selected pathway enrichment analysis method is **GlobalAncova**.

4.3 Pathway Topology Analysis

The structure of biological pathways represent our knowledge about the complex relationships among molecules within a cell or a living organism. However, most pathway analysis algorithms fail to take the structural information into consideration when estimating which pathways are significantly changed under conditions of study It is well-known that changes in more important positions of a network will trigger a more severe impact on the pathway than changes occured in marginal or relatively isolated positions.

The pathway toplogy analysis uses two well-established node centrality measures to estimate node importance - **degree centrality** and **betweenness centrality**. Degree centrality is defined as the number of links occured upon a node. For directed graph, there are two types of degree: in-degree for links come from other nodes, and out-degree for links initiated from the current node. Metabolic networks are directed graph. Here we only consider the out-degree for node importance measure. It is assumed that nodes in upstream will have regulatory roles for the downstream nodes, not vice versa. The betweenness centrality measures number of shortest paths going through the node. Since metabolic network is directed, we use relative betweenness centrality for metabolite importance measure. The degree centrality measures focus more on local connectivities, while the betweenness centrality measures focus more on global network topology. For more detailed discussions on various graph-based methods for analysing biological networks, please refer to the article by Tero Aittokallio, T. et al. [3]

Please note, for comparison among different pathways, the node importance values calculated from centrality measures are further normalized by the sum of the importance of the pathway. Therefore, the total/maximum importance of each pathway is 1; the importance measure of each metabolite node is actually the percentage w.r.t the total pathway importance, and the pathway impact value is the cumulative percentage from the matched metabolite nodes.

Your selected node importance measure for topological analysis is **relative betweenness centrality**.

5 Pathway Analysis Result

The results from pathway analysis are presented graphically as well as in a detailed table.

A Google-map style interactive visualization system was implemented to facilitate data exploration. The graphical output contains three levels of view: **metabolome view**, **pathway view**, and **compound view**. Only the metabolome view is shown below. Pathway views and compound views are generated dynamically based on your interactions with the visualization system. They are available in your downloaded files.

[2]Manuela Hummel, Reinhard Meister and Ulrich Mansmann. *GlobalANCOVA: exploration and assessment of gene group effects*, Bioinformatics 2008 24(1):78-85

[3]Tero Aittokallio and Benno Schwikowski. *Graph-based methods for analysing networks in cell biology*, Briefings in Bioinformatics 2006 7(3):243-255

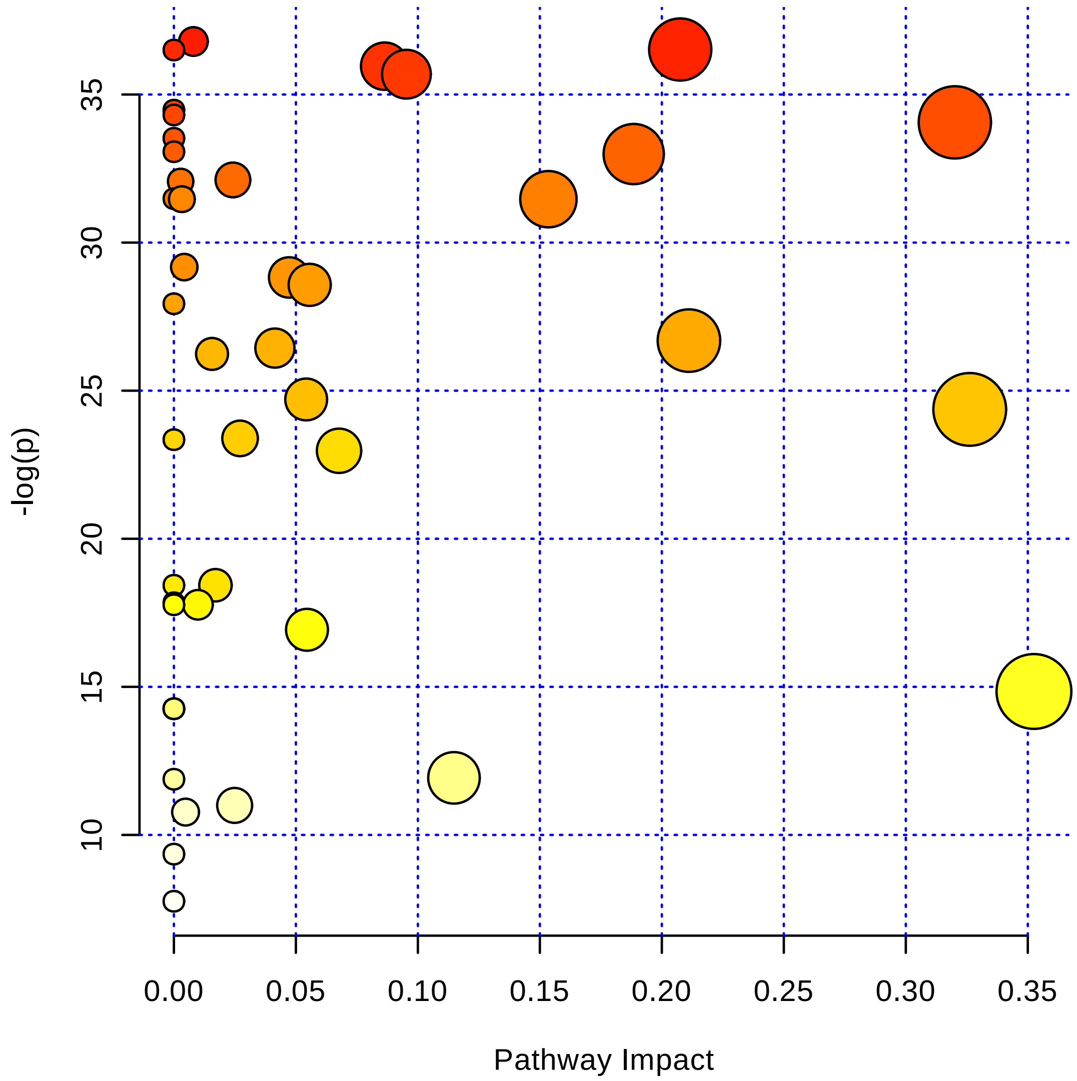

Figure 1: Summary of Pathway Analysis

The table below shows the detailed results from the pathway analysis. Since we are testing many pathways at the same time, the statistical p values from enrichment analysis are further adjusted for multiple testings. In particular, the **Total** is the total number of compounds in the pathway; the **Hits** is the actually matched number from the user uploaded data; the **Raw p** is the original p value calculated from the enrichment analysis; the **Holm p** is the p value adjusted by Holm-Bonferroni method; the **FDR p** is the p value adjusted using False Discovery Rate; the **Impact** is the pathway impact value calculated from pathway topology analysis.

Table 2: Result from Pathway Analysis

	Total Cmpd	Hits	Raw p	-log(p)	Holm adjust	FDR	Impact
Arginine and proline metabolism	77	4	0.00E+00	Inf	0.00E+00	0.00E+00	0.22
Phenylalanine metabolism	45	6	0.00E+00	Inf	0.00E+00	0.00E+00	0.20
Tryptophan metabolism	79	1	0.00E+00	Inf	0.00E+00	0.00E+00	0.01
Nitrogen metabolism	39	5	0.00E+00	Inf	0.00E+00	0.00E+00	0.00
Phenylalanine, tyrosine and tryptophan biosynthesis	27	2	1.05E-16	3.68E+01	4.74E-15	9.83E-16	0.01
Histidine metabolism	44	2	1.38E-16	3.65E+01	6.07E-15	9.83E-16	0.21
Aminoacyl-tRNA biosynthesis	75	7	1.40E-16	3.65E+01	6.07E-15	9.83E-16	0.00
Pentose phosphate pathway	32	3	2.42E-16	3.60E+01	1.02E-14	1.48E-15	0.09
Glycolysis or Gluconeogenesis	31	3	3.17E-16	3.57E+01	1.30E-14	1.73E-15	0.10
Thiamine metabolism	24	2	1.07E-15	3.45E+01	4.28E-14	5.24E-15	0.00
beta-Alanine metabolism	28	1	1.26E-15	3.43E+01	4.90E-14	5.60E-15	0.00
Pyruvate metabolism	32	2	1.61E-15	3.41E+01	6.13E-14	6.59E-15	0.32
Glutathione metabolism	38	2	2.76E-15	3.35E+01	1.02E-13	1.04E-14	0.00
D-Arginine and D-ornithine metabolism	8	1	4.35E-15	3.31E+01	1.57E-13	1.52E-14	0.00
Glycerolipid metabolism	32	2	4.71E-15	3.30E+01	1.65E-13	1.54E-14	0.19
Ascorbate and aldarate metabolism	45	2	1.13E-14	3.21E+01	3.83E-13	3.42E-14	0.02
Galactose metabolism	41	2	1.19E-14	3.21E+01	3.91E-13	3.42E-14	0.00
Ubiquinone and other terpenoid-quinone biosynthesis	36	1	2.12E-14	3.15E+01	6.80E-13	5.29E-14	0.00
Citrate cycle (TCA cycle)	20	2	2.16E-14	3.15E+01	6.80E-13	5.29E-14	0.15
Glyoxylate and dicarboxylate metabolism	50	2	2.16E-14	3.15E+01	6.80E-13	5.29E-14	0.00
Purine metabolism	92	2	2.14E-13	2.92E+01	6.21E-12	4.99E-13	0.00
Tyrosine metabolism	76	2	3.04E-13	2.88E+01	8.50E-12	6.76E-13	0.05
Valine, leucine and isoleucine biosynthesis	27	4	3.90E-13	2.86E+01	1.05E-11	8.31E-13	0.06
Propanoate metabolism	35	2	7.38E-13	2.79E+01	1.92E-11	1.51E-12	0.00
Glycine, serine and threonine metabolism	48	3	2.56E-12	2.67E+01	6.40E-11	5.02E-12	0.21
Valine, leucine and isoleucine degradation	40	3	3.30E-12	2.64E+01	7.91E-11	6.21E-12	0.04
Lysine degradation	47	3	4.01E-12	2.62E+01	9.23E-11	7.28E-12	0.02
Pyrimidine metabolism	60	1	1.87E-11	2.47E+01	4.11E-10	3.27E-11	0.05
D-Glutamine and D-glutamate metabolism	11	1	2.61E-11	2.44E+01	5.49E-10	4.41E-11	0.33
Vitamin B6 metabolism	32	2	6.96E-11	2.34E+01	1.39E-09	1.14E-10	0.03
Cyanoamino acid metabolism	16	2	7.26E-11	2.33E+01	1.39E-09	1.15E-10	0.00
Lysine biosynthesis	32	1	1.06E-10	2.30E+01	1.90E-09	1.62E-10	0.07
Starch and sucrose metabolism	50	1	9.90E-09	1.84E+01	1.68E-07	1.43E-08	0.02
Amino sugar and nucleotide sugar metabolism	88	1	9.90E-09	1.84E+01	1.68E-07	1.43E-08	0.00
Pantothenate and CoA biosynthesis	27	2	1.79E-08	1.78E+01	2.68E-07	2.50E-08	0.00
Glycerophospholipid metabolism	39	1	1.91E-08	1.78E+01	2.68E-07	2.54E-08	0.01
Ether lipid metabolism	23	1	1.91E-08	1.78E+01	2.68E-07	2.54E-08	0.00
Cysteine and methionine metabolism	56	2	4.46E-08	1.69E+01	5.35E-07	5.74E-08	0.05
Taurine and hypotaurine metabolism	20	2	3.58E-07	1.48E+01	3.93E-06	4.49E-07	0.35
Pentose and glucuronate interconversions	53	1	6.40E-07	1.43E+01	6.40E-06	7.29E-07	0.00
Alanine, aspartate and glutamate metabolism	24	1	6.40E-07	1.43E+01	6.40E-06	7.29E-07	0.00
Nicotinate and nicotinamide metabolism	44	1	6.40E-07	1.43E+01	6.40E-06	7.29E-07	0.00
Terpenoid backbone biosynthesis	33	1	6.40E-07	1.43E+01	6.40E-06	7.29E-07	0.00
Butanoate metabolism	40	2	6.61E-06	1.19E+01	3.97E-05	7.36E-06	0.11
Fatty acid biosynthesis	49	1	6.92E-06	1.19E+01	3.97E-05	7.54E-06	0.00
Primary bile acid biosynthesis	47	3	1.68E-05	1.10E+01	6.70E-05	1.78E-05	0.02
Fatty acid metabolism	50	1	2.10E-05	1.08E+01	6.70E-05	2.19E-05	0.00
Porphyrin and chlorophyll metabolism	104	1	8.68E-05	9.35E+00	1.74E-04	8.86E-05	0.00
Methane metabolism	34	2	4.26E-04	7.76E+00	4.26E-04	4.26E-04	0.00

The report was generated on Mon Aug 8 12:28:47 2016 with R version 3.2.2 (2015-08-14). For suggestions and feedback please contact Dr. Jeff Xia (*jeff.xia@mcgill.ca*).
