# Supplementary material for: Serum Metabolomics Analysis Reveals a Distinct Metabolic Profile of Patients with Primary Biliary Cholangitis
Source: Sci Rep. 2017 Apr 11;7:784. doi: 10.1038/s41598-017-00944-9 (PMC5429753; doi:10.1038/s41598-017-00944-9)
Supplement: Supplementary file 3 — Supplementary Material 3 [file 41598_2017_944_MOESM3_ESM.pdf]

Serum Metabolomics Analysis Reveals a Distinct Metabolic Profile of Patients with Primary Biliary Cholangitis

Juan Hao[1]. Tao Yang[1,2]. Yang Zhou[1], Guo-Yuan Gao[1,3], Feng Xing[1], Yuan Peng[1], Yan-Yan Tao[1], and Cheng-Hai Liu[1,4,5]

1 Institute of Liver Diseases, Shuguang Hospital Affiliated to Shanghai University of Traditional Chinese Medicine, 528 Zhangheng Road, Shanghai 201203, China

2 Institute of Cardiovascular Disease, Shuguang Hospital Affiliated to Shanghai University of Traditional Chinese Medicine, Shanghai 201203, China

3 School of Pharmacy, East China University of Science and Technology, Shanghai 200237, China

4 E-Institute of Traditional Chinese Medicine Internal Medicine, Shanghai Municipal Education Commission, 1200 Cailun Road, Shanghai 201203, China

5 Shanghai Key Laboratory of Traditional Chinese Clinical Medicine, Shanghai 201203, China

Correspondence author:

Cheng-Hai Liu, Institute of Liver Diseases, Shuguang Hospital Affiliated to Shanghai University of Traditional Chinese Medicine, 528 Zhangheng Road, Shanghai 201203, China; E-mail: chenghailiu@hotmail.com

Supplementary material

Supplementary material 3: Comparison of ROC curves and ROC curve of P4

Comparison of ROC curves

Variable 1	P1
Variable 2	P2
Variable 3	P3
Variable 4	P4
Classification variable	Group

Sample size		97
Positive group :	Group = 1	26
Negative group :	Group = 0	71

	AUC	SE [a]	95% CI [b]
P1	0.857	0.0373	0.771 to 0.920
P2	0.905	0.0296	0.829 to 0.955
P3	0.927	0.0257	0.856 to 0.970
P4	0.937	0.0227	0.868 to 0.976

[a] DeLong et al., 1988
[b] Binomial exact

ROC curve of P4

Variable	P4
Classification variable	Group

Sample size		97
Positive group :	Group = 1	26
Negative group :	Group = 0	71

Disease prevalence (%)	26.8

Area under the ROC curve (AUC)

Area under the ROC curve (AUC)	0.937
Standard Error[a]	0.0227
95% Confidence interval[b]	0.868 to 0.976
z statistic	19.258
Significance level P (Area=0.5)	<0.0001

[a] DeLong et al., 1988
[b] Binomial exact

Youden index

Youden index J	0.7541
Associated criterion	>-1.2129

Optimal criterion

Optimal criterion[a]	>0.0238
Sensitivity	69.23
Specificity	92.96

[a] Taking into account disease prevalence and estimated costs:
cost False Positive: 1; cost False Negative: 1
cost True Positive: 0; cost True Negative: 0

Criterion values and coordinates of the ROC curve

Criterio	Sensitivit	95% C	Specificit	95% C	+LR	-LR	+PV	-PV	Cost

n	y	l	y	l					
≥-6.4625	100.00	86.8 - 100.0	0.00	0.0 - 5.1	1.00		26.8		0.732
>-6.4625	100.00	86.8 - 100.0	1.41	0.04 - 7.6	1.01	0.00	27.1	100.0	0.722
>-6.2347	100.00	86.8 - 100.0	2.82	0.3 - 9.8	1.03	0.00	27.4	100.0	0.711
>-6.1145	100.00	86.8 - 100.0	4.23	0.9 - 11.9	1.04	0.00	27.7	100.0	0.701
>-6.0719	100.00	86.8 - 100.0	5.63	1.6 - 13.8	1.06	0.00	28.0	100.0	0.691
>-5.7563	100.00	86.8 - 100.0	7.04	2.3 - 15.7	1.08	0.00	28.3	100.0	0.680
>-5.5558	100.00	86.8 - 100.0	8.45	3.2 - 17.5	1.09	0.00	28.6	100.0	0.670
>-5.378	100.00	86.8 - 100.0	9.86	4.1 - 19.3	1.11	0.00	28.9	100.0	0.660
>-5.3576	100.00	86.8 - 100.0	11.27	5.0 - 21.0	1.13	0.00	29.2	100.0	0.649
>-5.3182	100.00	86.8 - 100.0	12.68	6.0 - 22.7	1.15	0.00	29.5	100.0	0.639
>-5.2783	100.00	86.8 - 100.0	14.08	7.0 - 24.4	1.16	0.00	29.9	100.0	0.629
>-5.2736	100.00	86.8 - 100.0	15.49	8.0 - 26.0	1.18	0.00	30.2	100.0	0.619
>-5.2678	100.00	86.8 - 100.0	16.90	9.0 - 27.7	1.20	0.00	30.6	100.0	0.608
>-5.132	100.00	86.8 - 100.0	18.31	10.1 - 29.3	1.22	0.00	31.0	100.0	0.598
>-5.0392	100.00	86.8 - 100.0	19.72	11.2 - 30.9	1.25	0.00	31.3	100.0	0.588
>-5.0374	100.00	86.8 - 100.0	21.13	12.3 - 32.4	1.27	0.00	31.7	100.0	0.577
>-4.8529	100.00	86.8 - 100.0	22.54	13.5 - 34.0	1.29	0.00	32.1	100.0	0.567
>-4.7486	100.00	86.8 - 100.0	23.94	14.6 - 35.5	1.31	0.00	32.5	100.0	0.557
>-4.7107	100.00	86.8 - 100.0	25.35	15.8 - 37.1	1.34	0.00	32.9	100.0	0.546
>-4.5945	100.00	86.8 - 100.0	26.76	16.9 - 38.6	1.37	0.00	33.3	100.0	0.536
>-4.4757	100.00	86.8 - 100.0	28.17	18.1 - 40.1	1.39	0.00	33.8	100.0	0.526
>-4.3654	100.00	86.8 - 100.0	29.58	19.3 - 41.6	1.42	0.00	34.2	100.0	0.515
>-4.2961	100.00	86.8 - 100.0	30.99	20.5 - 43.1	1.45	0.00	34.7	100.0	0.505
>-4.2325	100.00	86.8 - 100.0	32.39	21.8 - 44.5	1.48	0.00	35.1	100.0	0.495
>-4.2076	100.00	86.8 - 100.0	33.80	23.0 - 46.0	1.51	0.00	35.6	100.0	0.485
>-4.2047	100.00	86.8 - 100.0	35.21	24.2 - 47.5	1.54	0.00	36.1	100.0	0.474
>-4.169	100.00	86.8 - 100.0	36.62	25.5 - 48.9	1.58	0.00	36.6	100.0	0.464
>-	100.00	86.8 -	38.03	26.8 -	1.61	0.00	37.1	100.	0.45

4.1553		100.0		50.3				0	4
>-4.1149	100.00	86.8 - 100.0	39.44	28.0 - 51.7	1.65	0.00	37.7	100.0	0.443
>-4.1114	100.00	86.8 - 100.0	40.85	29.3 - 53.2	1.69	0.00	38.2	100.0	0.433
>-4.1047	100.00	86.8 - 100.0	42.25	30.6 - 54.6	1.73	0.00	38.8	100.0	0.423
>-4.0512	100.00	86.8 - 100.0	43.66	31.9 - 56.0	1.77	0.00	39.4	100.0	0.412
>-4.045	100.00	86.8 - 100.0	45.07	33.2 - 57.3	1.82	0.00	40.0	100.0	0.402
>-3.9462	100.00	86.8 - 100.0	46.48	34.5 - 58.7	1.87	0.00	40.6	100.0	0.392
>-3.7086	100.00	86.8 - 100.0	47.89	35.9 - 60.1	1.92	0.00	41.3	100.0	0.381
>-3.6013	100.00	86.8 - 100.0	49.30	37.2 - 61.4	1.97	0.00	41.9	100.0	0.371
>-3.451	100.00	86.8 - 100.0	50.70	38.6 - 62.8	2.03	0.00	42.6	100.0	0.361
>-3.3233	100.00	86.8 - 100.0	52.11	39.9 - 64.1	2.09	0.00	43.3	100.0	0.351
>-3.3096	100.00	86.8 - 100.0	53.52	41.3 - 65.5	2.15	0.00	44.1	100.0	0.340
>-3.3012	100.00	86.8 - 100.0	54.93	42.7 - 66.8	2.22	0.00	44.8	100.0	0.330
>-3.2802	100.00	86.8 - 100.0	56.34	44.0 - 68.1	2.29	0.00	45.6	100.0	0.320
>-3.0327	100.00	86.8 - 100.0	57.75	45.4 - 69.4	2.37	0.00	46.4	100.0	0.309
>-2.9158	100.00	86.8 - 100.0	59.15	46.8 - 70.7	2.45	0.00	47.3	100.0	0.299
>-2.8246	100.00	86.8 - 100.0	60.56	48.3 - 72.0	2.54	0.00	48.1	100.0	0.289
>-2.6953	100.00	86.8 - 100.0	61.97	49.7 - 73.2	2.63	0.00	49.1	100.0	0.278
>-2.6924	100.00	86.8 - 100.0	63.38	51.1 - 74.5	2.73	0.00	50.0	100.0	0.268
>-2.6483	100.00	86.8 - 100.0	64.79	52.5 - 75.8	2.84	0.00	51.0	100.0	0.258
>-2.567	100.00	86.8 - 100.0	66.20	54.0 - 77.0	2.96	0.00	52.0	100.0	0.247
>-2.564	100.00	86.8 - 100.0	67.61	55.5 - 78.2	3.09	0.00	53.1	100.0	0.237
>-2.4816	100.00	86.8 - 100.0	69.01	56.9 - 79.5	3.23	0.00	54.2	100.0	0.227
>-2.4625	100.00	86.8 - 100.0	70.42	58.4 - 80.7	3.38	0.00	55.3	100.0	0.216
>-2.4614	100.00	86.8 - 100.0	71.83	59.9 - 81.9	3.55	0.00	56.5	100.0	0.206
>-2.4023	100.00	86.8 - 100.0	73.24	61.4 - 83.1	3.74	0.00	57.8	100.0	0.196
>-2.1465	100.00	86.8 - 100.0	74.65	62.9 - 84.2	3.94	0.00	59.1	100.0	0.186
>-2.1225	96.15	80.4 - 99.9	74.65	62.9 - 84.2	3.79	0.052	58.1	98.1	0.196
>-	92.31	74.9 -	74.65	62.9 -	3.64	0.10	57.1	96.4	0.20

1.9321		99.1		84.2					6
>-1.7805	92.31	74.9 - 99.1	76.06	64.5 - 85.4	3.86	0.10	58.5	96.4	0.196
>-1.7273	92.31	74.9 - 99.1	77.46	66.0 - 86.5	4.10	0.099	60.0	96.5	0.186
>-1.6928	92.31	74.9 - 99.1	78.87	67.6 - 87.7	4.37	0.098	61.5	96.6	0.175
>-1.5963	92.31	74.9 - 99.1	80.28	69.1 - 88.8	4.68	0.096	63.2	96.6	0.165
>-1.5395	92.31	74.9 - 99.1	81.69	70.7 - 89.9	5.04	0.094	64.9	96.7	0.155
>-1.2129	92.31	74.9 - 99.1	83.10	72.3 - 91.0	5.46	0.093	66.7	96.7	0.144
>-1.1715	88.46	69.8 - 97.6	83.10	72.3 - 91.0	5.23	0.14	65.7	95.2	0.155
>-1.1192	88.46	69.8 - 97.6	84.51	74.0 - 92.0	5.71	0.14	67.6	95.2	0.144
>-1.0217	84.62	65.1 - 95.6	84.51	74.0 - 92.0	5.46	0.18	66.7	93.7	0.155
>-1.0066	84.62	65.1 - 95.6	85.92	75.6 - 93.0	6.01	0.18	68.7	93.8	0.144
>-0.9538	80.77	60.6 - 93.4	85.92	75.6 - 93.0	5.73	0.22	67.7	92.4	0.155
>-0.8755	80.77	60.6 - 93.4	87.32	77.3 - 94.0	6.37	0.22	70.0	92.5	0.144
>-0.6682	76.92	56.4 - 91.0	87.32	77.3 - 94.0	6.07	0.26	69.0	91.2	0.155
>-0.6481	76.92	56.4 - 91.0	88.73	79.0 - 95.0	6.83	0.26	71.4	91.3	0.144
>-0.6263	73.08	52.2 - 88.4	88.73	79.0 - 95.0	6.49	0.30	70.4	90.0	0.155
>-0.4156	69.23	48.2 - 85.7	88.73	79.0 - 95.0	6.14	0.35	69.2	88.7	0.165
>-0.1996	69.23	48.2 - 85.7	90.14	80.7 - 95.9	7.02	0.34	72.0	88.9	0.155
>0.0076	69.23	48.2 - 85.7	91.55	82.5 - 96.8	8.19	0.34	75.0	89.0	0.144
>0.0238	69.23	48.2 - 85.7	92.96	84.3 - 97.7	9.83	0.33	78.3	89.2	0.134
>0.2499	65.38	44.3 - 82.8	92.96	84.3 - 97.7	9.28	0.37	77.3	88.0	0.144
>0.4119	61.54	40.6 - 79.8	92.96	84.3 - 97.7	8.74	0.41	76.2	86.8	0.155
>0.6306	57.69	36.9 - 76.6	92.96	84.3 - 97.7	8.19	0.46	75.0	85.7	0.165
>0.7073	53.85	33.4 - 73.4	92.96	84.3 - 97.7	7.65	0.50	73.7	84.6	0.175
>0.7945	53.85	33.4 - 73.4	94.37	86.2 - 98.4	9.56	0.49	77.8	84.8	0.165
>0.8274	53.85	33.4 - 73.4	95.77	88.1 - 99.1	12.74	0.48	82.4	85.0	0.155
>0.8742	53.85	33.4 - 73.4	97.18	90.2 - 99.7	19.12	0.47	87.5	85.2	0.144
>0.9415	50.00	29.9 - 70.1	97.18	90.2 - 99.7	17.75	0.51	86.7	84.1	0.155
>1.0556	50.00	29.9 -	98.59	92.4 -	35.5	0.51	92.9	84.3	0.14

		70.1		100.0	0				4
>1.1996	46.15	26.6 - 66.6	98.59	92.4 - 100.0	32.77	0.55	92.3	83.3	0.155
>1.2429	46.15	26.6 - 66.6	100.00	94.9 - 100.0		0.54	100.0	83.5	0.144
>1.3407	42.31	23.4 - 63.1	100.00	94.9 - 100.0		0.58	100.0	82.6	0.155
>1.6309	38.46	20.2 - 59.4	100.00	94.9 - 100.0		0.62	100.0	81.6	0.165
>1.7104	34.62	17.2 - 55.7	100.00	94.9 - 100.0		0.65	100.0	80.7	0.175
>2.6489	30.77	14.3 - 51.8	100.00	94.9 - 100.0		0.69	100.0	79.8	0.186
>2.7282	26.92	11.6 - 47.8	100.00	94.9 - 100.0		0.73	100.0	78.9	0.196
>2.8208	23.08	9.0 - 43.6	100.00	94.9 - 100.0		0.77	100.0	78.0	0.206
>3.0899	19.23	6.6 - 39.4	100.00	94.9 - 100.0		0.81	100.0	77.2	0.216
>3.1407	15.38	4.4 - 34.9	100.00	94.9 - 100.0		0.85	100.0	76.3	0.227
>3.1874	11.54	2.4 - 30.2	100.00	94.9 - 100.0		0.88	100.0	75.5	0.237
>3.4854	7.69	0.9 - 25.1	100.00	94.9 - 100.0		0.92	100.0	74.7	0.247
>3.7129	3.85	0.10 - 19.6	100.00	94.9 - 100.0		0.96	100.0	74.0	0.258
>3.8036	0.00	0.0 - 13.2	100.00	94.9 - 100.0		1.00		73.2	0.268
