# Supplementary material for: Serum Metabolomics Analysis Reveals a Distinct Metabolic Profile of Patients with Primary Biliary Cholangitis
Source: Sci Rep. 2017 Apr 11;7:784. doi: 10.1038/s41598-017-00944-9 (PMC5429753; doi:10.1038/s41598-017-00944-9)
Supplement: Supplementary file 4 — Supplementary Material 4 [file 41598_2017_944_MOESM4_ESM.pdf]

# Serum Metabolomics Analysis Reveals a Distinct Metabolic Profile of Patients with Primary Biliary Cholangitis

Juan Hao[1]. Tao Yang[1,2]. Yang Zhou[1], Guo-Yuan Gao[1,3], Feng Xing[1], Yuan Peng[1], Yan-Yan Tao[1], and Cheng-Hai Liu[1,4,5]

1 Institute of Liver Diseases, Shuguang Hospital Affiliated to Shanghai University of Traditional Chinese Medicine, 528 Zhangheng Road, Shanghai 201203, China

2 Institute of Cardiovascular Disease, Shuguang Hospital Affiliated to Shanghai University of Traditional Chinese Medicine, Shanghai 201203, China

3 School of Pharmacy, East China University of Science and Technology, Shanghai 200237, China

4 E-Institute of Traditional Chinese Medicine Internal Medicine, Shanghai Municipal Education Commission, 1200 Cailun Road, Shanghai 201203, China

5 Shanghai Key Laboratory of Traditional Chinese Clinical Medicine, Shanghai 201203, China

**Correspondence author:**

Cheng-Hai Liu, Institute of Liver Diseases, Shuguang Hospital Affiliated to Shanghai University of Traditional Chinese Medicine, 528 Zhangheng Road, Shanghai 201203, China; E-mail: chenghailiu@hotmail.com

## Supplementary material

Supplementary material 4: The values of metabolites in the validation dataset and Comparison of ROC curves in validation dataset

The values of metabolites in the validation dataset

| ID | Group | Group1 | 4_Hydroxyproline | Pyruvate | Citraconate | 3_Hydroxyisovalerate | P4 |
|---|---|---|---|---|---|---|---|
| AIH1 | AIH | 0 | 0.031345 | 0.007714 | 0.003985 | 0.01193714 | -5.864311115 |
| AIH2 | AIH | 0 | 0.023823 | 0.012272 | 0.002768 | 0.01774285 | -4.692618164 |
| AIH3 | AIH | 0 | 0.030435 | 0.007526 | 0.004291 | 0.02251629 | -4.033450963 |
| AIH4 | AIH | 0 | 0.042922 | 0.003889 | 0.006852 | 0.01111855 | -5.984868738 |
| AIH5 | AIH | 0 | 0.032538 | 0.009906 | 0.004431 | 0.01471029 | -5.845184433 |
| AIH6 | AIH | 0 | 0.026444 | 0.010139 | 0.00384 | 0.01780659 | -4.870986162 |
| AIH7 | AIH | 0 | 0.024649 | 0.003843 | 0.004052 | 0.01521022 | -3.674487664 |
| HBC1 | HBC | 0 | 0.034434 | 0.005974 | 0.002413 | 0.0232238 | -3.138860451 |
| HBC2 | HBC | 0 | 0.022113 | 0.015151 | 0.002878 | 0.01731403 | -5.040021484 |
| HBC3 | HBC | 0 | 0.040412 | 0.004957 | 0.003082 | 0.01350283 | -4.889936574 |
| HBC4 | HBC | 0 | 0.034945 | 0.003802 | 0.003109 | 0.00837654 | -5.615381643 |
| HBC5 | HBC | 0 | 0.029489 | 0.005783 | 0.004977 | 0.01001332 | -6.038125063 |
| HBC6 | HBC | 0 | 0.037599 | 0.005982 | 0.003287 | 0.01595857 | -4.679385311 |
| HBC7 | HBC | 0 | 0.039482 | 0.010152 | 0.004401 | 0.01792105 | -5.601528345 |
| HBC8 | HBC | 0 | 0.029477 | 0.009521 | 0.00503 | 0.01319952 | -6.07708574 |
| HBC9 | HBC | 0 | 0.044672 | 0.003173 | 0.004795 | 0.02892558 | -2.581868294 |
| PBC1 | PBC | 1 | 0.025263 | 0.007158 | 0.002783 | 0.023158 | -3.13092769 |
| PBC10 | PBC | 1 | 0.020413 | 0.009549 | 0.002715 | 0.02584187 | -2.945039622 |
| PBC11 | PBC | 1 | 0.031576 | 0.004358 | 0.00392 | 0.03533565 | -1.795515418 |
| PBC12 | PBC | 1 | 0.031479 | 0.005656 | 0.002806 | 0.02015448 | -3.480844793 |
| PBC13 | PBC | 1 | 0.032089 | 0.005292 | 0.002748 | 0.03241024 | -2.012049119 |
| PBC14 | PBC | 1 | 0.011164 | 0.013197 | 0.00311 | 0.01501097 | -4.277538153 |
| PBC15 | PBC | 1 | 0.025861 | 0.006222 | 0.003918 | 0.03087678 | -2.470201298 |
| PBC16 | PBC | 1 | 0.024794 | 0.0076 | 0.004058 | 0.01543318 | -4.768234759 |
| PBC17 | PBC | 1 | 0.023594 | 0.008104 | 0.003868 | 0.02391477 | -3.489570952 |
| PBC18 | PBC | 1 | 0.036646 | 0.004731 | 0.003444 | 0.01938533 | -3.743473766 |
| PBC19 | PBC | 1 | 0.027798 | 0.004297 | 0.005163 | 0.04754507 | -1.019219473 |
| PBC2 | PBC | 1 | 0.024393 | 0.006349 | 0.003567 | 0.03935221 | -1.61895731 |
| PBC20 | PBC | 1 | 0.02957 | 0.004967 | 0.002484 | 0.01482156 | -3.927292783 |
| PBC22 | PBC | 1 | 0.023037 | 0.006207 | 0.00337 | 0.03728155 | -1.590889141 |
| PBC3 | PBC | 1 | 0.016368 | 0.014334 | 0.002508 | 0.01827608 | -4.195404226 |
| PBC4 | PBC | 1 | 0.021149 | 0.004638 | 0.003377 | 0.01071352 | -4.568801186 |
| PBC5 | PBC | 1 | 0.025162 | 0.002771 | 0.003539 | 0.02718111 | -1.352830425 |
| PBC6 | PBC | 1 | 0.021057 | 0.004277 | 0.002414 | 0.04429148 | 0.007369912 |
| PBC7 | PBC | 1 | 0.032113 | 0.005773 | 0.003855 | 0.01861011 | -4.11062884 |
| PBC8 | PBC | 1 | 0.033299 | 0.006224 | 0.003558 | 0.02224546 | -3.691209239 |
| PBC9 | PBC | 1 | 0.02 | 0.006844 | 0.00308 | 0.04418847 | -0.955672939 |

**Comparison of ROC curves in validation dataset**

| | |
|---|---|
| Variable1 | 3Hydroxyisovalerate |
| Variable2 | 4Hydroxyproline |
| Variable3 | Citraconate |
| Variable4 | Pyruvate |
| Variable5 | P4 |
| Classification variable | Group1 |

| | | |
|---|---|---|
| Sample size | | 37 |
| Positive group: | Group1=1 | 21 |
| Negative group: | Group1=0 | 16 |

| | AUC | SE [a] | 95% CI [b] |
|---|---|---|---|
| 3-Hydroxyisovalerate | 0.821 | 0.0699 | 0.661 to 0.927 |
| 4-Hydroxyproline | 0.768 | 0.0787 | 0.600 to 0.890 |
| Citraconate | 0.688 | 0.0958 | 0.514 to 0.829 |
| Pyruvate | 0.560 | 0.104 | 0.387 to 0.722 |
| P4 | 0.890 | 0.0550 | 0.743 to 0.969 |

[a] DeLong et al., 1988

[b] Binomial exact

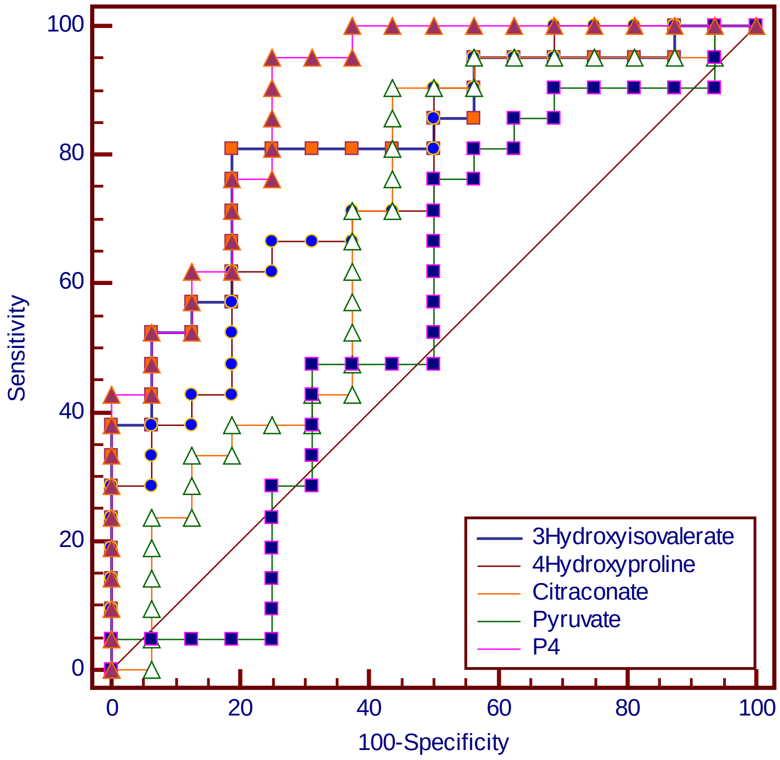

## ROC curve of P4

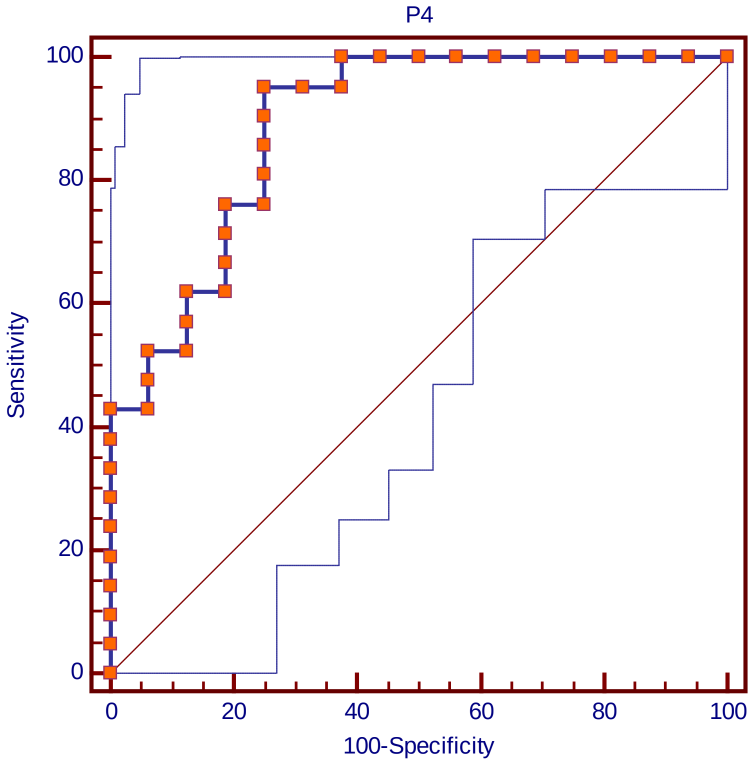


| | |
|---|---|
| Variable | P4 |
| Classification variable | Group1 |

| | | |
|---|---|---|
| Sample size | | 37 |
| Positive group: | Group1 = 1 | 21 |
| Negative group: | Group1 = 0 | 16 |

| | |
|---|---|
| Disease prevalence(%) | unknown |

### Area under the ROC curve (AUC)

| | |
|---|---|
| Area under the ROC curve (AUC) | 0.890 |
| Standard Error[a] | 0.0550 |
| 95% Confidence interval[b] | 0.743 to 0.969 |
| Z statistic | 7.089 |
| Significance level P (Area=0.5) | <0.0001 |

[a] DeLong et al., 1988
[b] Binomial exact

### Youden index

| | |
|---|---|
| Youden index J | 0.7024 |
| Associated criterion | >-4.6794 |

### Criterion values and coordinates of the ROC curve

| Criterion | Sensitivity | 95% CI | Specificity | 95% CI | +LR | -LR |
|---|---|---|---|---|---|---|
| ≥-6.0771 | 100.00 | 83.9 - 100.0 | 0.00 | 0.0 - 20.6 | 1.00 | |
| >-6.0771 | 100.00 | 83.9 - 100.0 | 6.25 | 0.2 - 30.2 | 1.07 | 0.00 |
| >-6.0381 | 100.00 | 83.9 - 100.0 | 12.50 | 1.6 - 38.3 | 1.14 | 0.00 |
| >-5.9849 | 100.00 | 83.9 - 100.0 | 18.75 | 4.0 - 45.6 | 1.23 | 0.00 |
| >-5.8643 | 100.00 | 83.9 - 100.0 | 25.00 | 7.3 - 52.4 | 1.33 | 0.00 |
| >-5.8452 | 100.00 | 83.9 - 100.0 | 31.25 | 11.0 - 58.7 | 1.45 | 0.00 |
| >-5.6154 | 100.00 | 83.9 - 100.0 | 37.50 | 15.2 - 64.6 | 1.60 | 0.00 |
| >-5.6015 | 100.00 | 83.9 - 100.0 | 43.75 | 19.8 - 70.1 | 1.78 | 0.00 |
| >-5.04 | 100.00 | 83.9 - 100.0 | 50.00 | 24.7 - 75.3 | 2.00 | 0.00 |
| >-4.8899 | 100.00 | 83.9 - 100.0 | 56.25 | 29.9 - 80.2 | 2.29 | 0.00 |
| >-4.871 | 100.00 | 83.9 - 100.0 | 62.50 | 35.4 - 84.8 | 2.67 | 0.00 |
| >-4.7682 | 95.24 | 76.2 - 99.9 | 62.50 | 35.4 - 84.8 | 2.54 | 0.076 |
| >-4.6926 | 95.24 | 76.2 - 99.9 | 68.75 | 41.3 - 89.0 | 3.05 | 0.069 |
| >-4.6794 | 95.24 | 76.2 - 99.9 | 75.00 | 47.6 - 92.7 | 3.81 | 0.063 |
| >-4.5688 | 90.48 | 69.6 - 98.8 | 75.00 | 47.6 - 92.7 | 3.62 | 0.13 |
| >-4.2775 | 85.71 | 63.7 - 97.0 | 75.00 | 47.6 - 92.7 | 3.43 | 0.19 |
| >-4.1954 | 80.95 | 58.1 - 94.6 | 75.00 | 47.6 - 92.7 | 3.24 | 0.25 |
| >-4.1106 | 76.19 | 52.8 - 91.8 | 75.00 | 47.6 - 92.7 | 3.05 | 0.32 |
| >-4.0335 | 76.19 | 52.8 - 91.8 | 81.25 | 54.4 - 96.0 | 4.06 | 0.29 |
| >-3.9273 | 71.43 | 47.8 - 88.7 | 81.25 | 54.4 - 96.0 | 3.81 | 0.35 |
| >-3.7435 | 66.67 | 43.0 - 85.4 | 81.25 | 54.4 - 96.0 | 3.56 | 0.41 |
| >-3.6912 | 61.90 | 38.4 - 81.9 | 81.25 | 54.4 - 96.0 | 3.30 | 0.47 |
| >-3.6745 | 61.90 | 38.4 - 81.9 | 87.50 | 61.7 - 98.4 | 4.95 | 0.44 |
| >-3.4896 | 57.14 | 34.0 - 78.2 | 87.50 | 61.7 - 98.4 | 4.57 | 0.49 |
| >-3.4808 | 52.38 | 29.8 - 74.3 | 87.50 | 61.7 - 98.4 | 4.19 | 0.54 |
| >-3.1389 | 52.38 | 29.8 - 74.3 | 93.75 | 69.8 - 99.8 | 8.38 | 0.51 |
| >-3.1309 | 47.62 | 25.7 - 70.2 | 93.75 | 69.8 - 99.8 | 7.62 | 0.56 |
| >-2.945 | 42.86 | 21.8 - 66.0 | 93.75 | 69.8 - 99.8 | 6.86 | 0.61 |
| >-2.5819 | 42.86 | 21.8 - 66.0 | 100.00 | 79.4 - 100.0 | | 0.57 |
| >-2.4702 | 38.10 | 18.1 - 61.6 | 100.00 | 79.4 - 100.0 | | 0.62 |
| >-2.012 | 33.33 | 14.6 - 57.0 | 100.00 | 79.4 - 100.0 | | 0.67 |
| >-1.7955 | 28.57 | 11.3 - 52.2 | 100.00 | 79.4 - 100.0 | | 0.71 |
| >-1.619 | 23.81 | 8.2 - 47.2 | 100.00 | 79.4 - 100.0 | | 0.76 |
| >-1.5909 | 19.05 | 5.4 - 41.9 | 100.00 | 79.4 - 100.0 | | 0.81 |
| >-1.3528 | 14.29 | 3.0 - 36.3 | 100.00 | 79.4 - 100.0 | | 0.86 |
| >-1.0192 | 9.52 | 1.2 - 30.4 | 100.00 | 79.4 - 100.0 | | 0.90 |
| >-0.9557 | 4.76 | 0.1 - 23.8 | 100.00 | 79.4 - 100.0 | | 0.95 |
| >0.0074 | 0.00 | 0.0 - 16.1 | 100.00 | 79.4 - 100.0 | | 1.00 |
